# Genetic parameters and genome-wide association for milk production traits and somatic cell score in different lactation stages of Shanghai Holstein population

**DOI:** 10.3389/fgene.2022.940650

**Published:** 2022-09-05

**Authors:** Dengying Liu, Zhong Xu, Wei Zhao, Shiyi Wang, Tuowu Li, Kai Zhu, Guanglei Liu, Xiaoduo Zhao, Qishan Wang, Yuchun Pan, Peipei Ma

**Affiliations:** ^1^ Shanghai Key Laboratory of Veterinary Biotechnology, Department of Animal Science, School of Agriculture and Biology, Shanghai Jiao Tong University, Shanghai, China; ^2^ Hubei Key Laboratory of Animal Embryo and Molecular Breeding, Institute of Animal Husbandry and Veterinary, Hubei Provincial Academy of Agricultural Sciences, Wuhan, China; ^3^ Shanghai Dairy Cattle Breeding Centre Co, Ltd, Shanghai, China; ^4^ Department of Animal Breeding and Reproduction, College of Animal Science, Zhejiang University, Hangzhou, China

**Keywords:** Shanghai Holstein population, milk production traits, genetic parameter, genome-wide association study, different stages of lactation

## Abstract

The aim of this study was to investigate the genetic parameters and genetic architectures of six milk production traits in the Shanghai Holstein population. The data used to estimate the genetic parameters consisted of 1,968,589 test-day records for 305,031 primiparous cows. Among the cows with phenotypes, 3,016 cows were genotyped with Illumina Bovine SNP50K BeadChip, GeneSeek Bovine 50K BeadChip, GeneSeek Bovine LD BeadChip v4, GeneSeek Bovine 150K BeadChip, or low-depth whole-genome sequencing. A genome-wide association study was performed to identify quantitative trait loci and genes associated with milk production traits in the Shanghai Holstein population using genotypes imputed to whole-genome sequences and both fixed and random model circulating probability unification and a mixed linear model with rMVP software. Estimated heritabilities (h2) varied from 0.04 to 0.14 for somatic cell score (SCS), 0.07 to 0.22 for fat percentage (FP), 0.09 to 0.27 for milk yield (MY), 0.06 to 0.23 for fat yield (FY), 0.09 to 0.26 for protein yield (PY), and 0.07 to 0.35 for protein percentage (PP), respectively. Within lactation, genetic correlations for SCS, FP, MY, FY, PY, and PP at different stages of lactation estimated in random regression model were ranged from -0.02 to 0.99, 0.18 to 0.99, 0.04 to 0.99, 0.04 to 0.99, 0.01 to 0.99, and 0.33 to 0.99, respectively. The genetic correlations were highest between adjacent DIM but decreased as DIM got further apart. Candidate genes included those related to production traits (*DGAT1*, *MGST1*, *PTK2*, and *SCRIB*), disease-related (*LY6K*, *COL22A1*, *TECPR2*, and *PLCB1*), heat stress–related (*ITGA9*, *NDST4*, *TECPR2*, and *HSF1*), and reproduction-related (*7SK* and *DOCK2*) genes. This study has shown that there are differences in the genetic mechanisms of milk production traits at different stages of lactation. Therefore, it is necessary to conduct research on milk production traits at different stages of lactation as different traits. Our results can also provide a theoretical basis for subsequent molecular breeding, especially for the novel genetic loci.

## Introduction

Chinese Holstein cattle are derived from grading crossbreeding and selection between the local yellow cattle and Holstein, a breed that was mostly imported from Canada, the United States, France, and northern Europe and renamed by the Chinese Ministry of Agriculture in 1992 (Huang et al., 2010; Ferreri et al., 2011). Since then, China has continuously imported live proven cattle, frozen semen, and embryos from most temperate countries for use in crossbreeding aimed at improving the productivity of Chinese native cattle by combining the environmental adaptation features of Chinese cattle with the high milk yield (MY) potential of foreign cattle (Ferreri et al., 2011; Zhang and Sun, 2021). Therefore, the genetic architecture of the Chinese Holstein population is different from other populations. China occupies a larger area and a larger span of north–south latitudes. Accordingly, topography, climate, herd management system, and other environments vary greatly in different regions, and the different climatic zones have differential contributions to population genetic characteristics, with Holstein in different countries or provinces having its own genetic characteristics (Pérez-Cabal et al., 2012; Liu et al., 2019). The Shanghai Holstein cattle population is raised under a subtropical environment and an intensive pasture system that is maintained below the level of severe thermal stress throughout the day in the summer season. At the same time, Shanghai is the main center for providing Holstein semen to various farms throughout China. Currently, Shanghai Holstein cattle are susceptible to mastitis. The average number of lactations for Shanghai Holstein cattle was 2.23, which makes it difficult to maintain production efficiency and meet the demands of the dairy industry, and the MY is much less than that in the United States (Mao, 2015; Liu et al., 2021).

Since 1994, the Dairy Herd Improvement has been carried out in Shanghai, where millions of test day records are collected (Sun et al., 2008). Milk production and quality, including MY, fat yield (FY), fat percentage (FP), protein yield (PY), protein percentage (PP), and somatic cell score (SCS), are the most important traits in the dairy industry. There are complex traits influenced by management practices and environmental conditions and the physiological stages (e.g., age and stage of lactation) and genetic merits of the animals. Genetic parameters such as heritability are the core of breeding work to accelerate genetic progress and also the most important properties of a population (Meyer, 1989; Akanno and Ibe, 2005). Evaluating genetic parameters is the basis for research such as genome-wide association study (GWAS) and genome-wide selection. However, the heritability of a phenotype in GWAS is too low, resulting in the reduced possibility of detecting the actual association between single nucleotide polymorphisms (SNPs) and traits or non-detection (Shao et al., 2021). Recently, there has been considerable interest in using the random regression model (RRM) to model individual test-day records for the genetic evaluation of milk traits (Khanzadeh et al., 2013; Silva et al., 2020; Soumri et al., 2020).

GWAS is an effective method for identifying the genetic variations involved in complex traits. With the rapid development of high-throughput sequencing technology, many researchers have reported that the power of GWAS based on imputed whole-genome sequencing (WGS) variants on different traits in livestock, such as cattle (Sanchez et al., 2017), pig (Wu et al., 2019; Yan et al., 2021), and chicken (Ye et al., 2020), was improved. Compared to microarray, WGS data cover all SNPs, including causative mutations. However, sequencing thousands of individuals of interest is expensive. Imputation from SNP panels to WGS data is an attractive and less expensive approach to obtain WGS data. Selection of the imputation reference panel is very important for genomic prediction with imputed WGS data. Nowadays, numerous GWASs are conducted on cattle by using the 1000 Bull Genomes Project to impute WGS data on genotyped animals (Iheshiulor et al., 2016; Meuwissen et al., 2021).

Thus far, many researchers have studied the Holstein population in different countries and provinces, including the north of China (Ferreri et al., 2011; Jiang et al., 2012; Liu et al., 2020; Silva et al., 2020). A previous study of the Shanghai Holstein population used the genotyping by genome reducing and sequencing (GGRS) of 1,092 cattle and revealed some SNPs associated with MY, FP, PP, and SCS (Chen Z. et al., 2018), but the study had a small sample size and only conducted association analysis of part of milk production traits using GGRS data. The use of imputed WGS data has been shown that can increase GWAS power and ability to detect causal mutations of complex traits. Therefore, the aim of the present study was to estimate the genetic parameters for milk production and quality traits by using RRM and find new genetic loci by using imputed WGS and a much larger population. In this study, we emphasized the different physiological stages of the mammary gland across the lactation stage. To the best of our knowledge, this is the first time that a GWAS for milk production traits was conducted using imputed WGS data in the south of China, where the Holstein population is suffering heavy heat stress.

## Material and methods

### Data

To evaluate the genetic parameter of milk production traits, we collected the test-day records from the farms of Shanghai Bright Dairy and Food Co., Ltd. from primiparous cows born between 1995 and 2020 with the regular and standard performance of DHI. In total, there are 1,968,589 records for the first lactation of 305,031 cows from 260 farms with the following criteria (Aerts et al., 2021; Mbuthia et al., 2021): 1) age at first calving between 19 and 37 months; 2) test day from 5 to 305 DIM, of which only 12% records out of the range; 3) milk yield of 1.0–65 kg, fat percentage of 0.5–8.5%, protein percentage of 0.5–7.5%, SCC less than 2 million cells per milliliter (Yang et al., 2013); 4) a minimum of three test-day records were required for a cow observation to be included in the analysis (Soumri et al., 2020), of which one was before DIM 45 (Bignardi et al., 2009); 5) the calving date was required to be before December 2019 so that all cows had the opportunity to finish the complete first lactation. A summary of data set used in this analysis is given in Table 1. The somatic cell count (SCC) was log-transformed in SCS as follows: SCS = log2 (SCC/100) + 3; FY was calculated as (FP*MY)/100; PY was calculated as (PP*MY)/100. The distribution of phenotypes is illustrated in Supplementary Figure S1. DMU Trace program was used for tracing ancestors and creating the full pedigree of the animals (Madsen, 2012). The pedigree was built by tracing the ancestors back as far as possible by using the sire-dam structure. Consequently, the pedigrees included 529,011 animals in total, which was recorded during the 1985–2019 period, including 4,945 sires and 19,867 dams, respectively. The inbreeding coefficients for the individuals with test-day records were calculated by going back only three generations in the pedigree. This data set included 226,602 animals. Estimates of the inbreeding coefficient were obtained using the R package “nadiv” (Wolak, 2012).

**TABLE 1:** Descriptive statistics of milk production and quality traits in Shanghai Holstein population.

Traits	No. of records	No. of animals	Mean	Standard deviation	Minimum	Maximum	CV
Milk yield (MY, kg/d)	1,859,464	240,681	27.80	8.35	0.1	300	0.30
Fat yield (FY, kg/d)	1,855,585	240,678	0.998	0.36	0.01	7.99	0.36
Protein yield (PY, kg/d)	1,843,598	240,680	0.866	0.25	0.003	6.944	0.29
Fat (FP, %)	1842807	240,679	3.64	0.88	0.02	15.90	0.24
Protein (PP, %)	1843717	240,681	3.15	0.38	0.1	15.90	0.12
SCS	1668583	240240	2.84	1.95	0.00	9.00	0.59

### Random regression test-day model

The derivative-free approach to multivariate analysis (DMU) package was used to estimate breeding values using the random regression test-day model (RRM) (Jakobsen et al., 2002a; Schaeffer, 2004). Due to problems with convergence, single trait RRM was used to estimate the genetic parameters for different traits. We considered herd-test date, calving month–age, and calving year–season as fixed effects, and individual additive genetic effects and permanent environment effects as random regression effects (Liu et al., 2020). Both random regressions were modeled using fifth-order Legendre polynomial. The model equation is as follows:

$$Y_{ijklmn}=HTD_{i}+Age_{j}+CDSD_{k}+\overset{5}{\underset{m=0}{\sum }}a_{lm}X_{m}\left(\omega \right)+\overset{5}{\underset{m=0}{\sum }}p_{lm}X_{m}\left(\omega \right)+e_{ijklmn}$$

Here, $Y_{ijklmn}$ is the test-day records; $HTD_{i}$ is the fixed effect of the *i*th herd-test day; $Age_{j}$ is the fixed effect of *j*th calving month–age; $CDSD_{k}$ is the fixed effect of the *k*th calving year–season; $a_{lm}$ is random regression coefficient for additive genetic effects specific to cow *l*; $p_{lm}$ is random regression coefficient for permanent environment effects specific to cow *l*; $X_{m}\left(\omega \right)$ is the *m*th covariate of Lengendre polynomial; ω is the days of lactation after standardization; and $e_{ijklmn}$ is the random residual effects.

The variance-covariance matrix is as follows:

$$Var\left[\begin{array}{c} a\\ p\\ e \end{array}\right]=\left[\begin{array}{ccc} G⊗A & 0 & 0\\ 0 & I⊗P & 0\\ 0 & 0 & R \end{array}\right]$$

Here, a is additive genetic random regression coefficient vector; p is permanent environment random regression coefficient vector; G is the variance–covariance matrix of additive genetic random regression coefficient; A is the numerator relationship matrix; P is the variance–covariance matrix of permanent environment random regression coefficient; I is the identity matrix; and R is the diagonal matrix of residual variance $\left(I\sigma_{e}^{2}\right)$ , which hypothesized the residuals are homogeneous. The homogeneous option dramatically reduces computing time without sacrifice as there is a minimal difference between the homogeneous model and the heterogenous model (López-Romero and Carabaño, 2003; Li J. et al., 2020).

### Genotyping, quality control, and imputation

Data from 3,489 genotyped animals were used in this study. In addition, 222 bulls from Run 2 of the 1000 Bull Genome Project were included (Daetwyler et al., 2014). The 3,489 animals were genotyped using different panels: GGP Bovine 50K chip (47,843 SNPs, GeneSeek Genomic Profiler, Neogen Corp., Lincoln, NE, United States, n = 294), GGP Bovine 150 K chip (140,668 SNPs, n = 1,744), GGP Bovine LD v4 (30,108 SNPs, n = 145), Illumina Bovine SNP50K v2 (54,609 SNPs, Illumina, San Diego, CA, United States, n = 1,100) and the extremely low-coverage whole genome sequencing with coverage at 0.5–1× (n = 206).

The extremely low-coverage whole genome sequencing used the Illumina Hiseq4000 platform to sequence the genomic DNA extracted from cow hair-follicle according to the manufacturer’s protocol. All of the raw sequence data were filtered using Fastp v0.20.0 (Chen S. et al., 2018) with default parameters and were then aligned to the pig genome build UMD3.1 using BWA mem algorithm implemented in samtools v1.10 (Li and Durbin, 2010). After removing PCR duplicates by Picard Tools v2.0.1 (http://broadinstitute.github.io/picard/), local realignment around indels and base quality scores recalculation were conducted using GATK v3.6 (McKenna et al., 2010) based on known indels and SNPs from in dbSNP database build 152. Sequenced individuals (n = 206) were used to carry out SNP calling *via* both bcftools v1.9 (Li, 2011) (set 1) and GATK UnifiedGenotyper (set 2), simultaneously. The overlapping SNPs between set 1 and set 2 were further filtered *via* GATK VQSR using known variants from the dbSNP database. Finally, a total of 12,396,463 autosomal SNPs with PASS flag and minor allele frequency (MAF) larger than 0.05 were retained. STITCH v1.5.3 (Davies et al., 2016) was used to impute the missing genotypes of the extremely low-coverage whole genome sequencing.

For all the genotype data, only the autosomal chromosomes and SNPs with known positions in the UMD 3.1 bovine assembly map were considered. Genotype quality control for all the panels excluded SNPs with a call rate lower than 0.90, SNPs with deviations from the Hardy–Weinberg equilibrium (*p* < 10^–6^) as calculated by means of the Fisher’s Exact Test, and SNPs with MAF lower than 0.05. For the quality control of the samples, animals with a call rate lower than 0.95 were excluded from the analysis.

The imputation of WGS genotypes from LD and 50K was performed in two steps. First, the LD and 50K genotypes were imputed to 150K, respectively. Then, in the second step, all imputed and real 150K genotypes were imputed to sequence data using 222 bulls from Run 2 of the 1000 Bull Genome Project (Daetwyler et al., 2014) and the UMD3.1 reference sequence. All the abovementioned steps used BEAGLE v4.1 (Browning and Browning, 2009) software. For the imputed extremely low-coverage whole genome sequencing, we used BEAGLE v4.1 to impute to WGS genotypes using 222 bulls as reference sequence described earlier.

All the genotypes imputed to WGS were merged using “bcftools merge--force-samples” (v1.3). We used Perl script to match phenotype samples ID with genotype samples ID to obtain the genotype file which has phenotype. Finally, genotype data were filtered by PLINK v1.9 with the parameters “--geno 0.1 --hwe 0.000001 --maf 0.05 --mind 0.05”. Only autosomal SNPs were considered in this study, and IDs without phenotypes were excluded.

### Principal component analysis

To determine the level of population stratification, we plotted the population structure by PCA. Principal component analysis (PCA) was conducted using GCTA v64 (Yang et al., 2011) on 3,016 cows genotyped with 8,686,483 markers covering the whole genome to study the population structure. The first two eigenvectors are selected to make a scatter plot, and according to the results of the scatter plot, it can be known whether the population is divided into several subgroups.

### GWAS analysis

We performed powerful GWAS analyses of six milk production traits (MY, FP, FY, PP, PY, and SCS) in different lactation stages (early lactation [TD7], peak lactation [TD35 and TD50], mid lactation [TD140], and late lactation [TD280]) in the Shanghai Holstein population using FarmCPU (Fixed and random model Circuitous Probability Unification) and MLM (mixed linear model) based on imputed WGS data with the rMVP software (Yin et al., 2021). FarmCPU method is a multi-locus linear mixed model which implements marker tests with associated markers as covariates in a fixed effect model and optimization on the associated covariate markers in a random effect model separately (Liu et al., 2016). As is known, population stratification is an important factor that can cause false positives in association studies. Therefore, the present study fitted the first three principal components (PCs) as covariate variables in the GWAS models to adjust for the population stratification. The model can be written as follows:

$$y=Tw+Pq+m_{k}h_{k}+e$$

Here, y is the vector of EBVs of individual; w is a matrix of fixed effect for the top three PCs; q is the pseudo quantitative trait nucleotides (QTNs) effects, which was used as the fixed effects, initiated as an empty set; T and P are the corresponding design matrices for w and q , respectively; $m_{k}$ is the genotype of the k marker; $h_{k}$ is the corresponding ; and e is the vector of residuals with assuming $e∼N\left(0,I\sigma_{e}^{2}\right)$ . The random effect model was used to select the most appropriate pseudo QTNs. The model can be written as follows:

y=u+e

Here, y is the vector of EBVs of individual; u is the genetic effect of the individual, and $u∼N\left(0,2K\sigma_{u}^{2}\right)$ , in which K is the kinship matrix derived from the pseudo QTNs, and $\sigma_{u}^{2}$ is an unknown genetic variance; and e is the residual effect vector.

### The MLM can be written as follows:

y=Wb+Zc+Sa+e

Here y is the vector of EBVs of individual; c is the vector of the same fixed effects as in the FarmCPU model; b is the vector of the SNP substitution effects, and a is the vector of random additive genetic effects with $a∼N\left(0,G\sigma_{a}^{2}\right)$ , where G is the genomic relationship matrix, and $\sigma_{a}^{2}$ is the additive variance. W , Z , and S are the incidence matrices for b , a , and c , respectively.

As suggested by Ji et al. (2019), we used 5 × 10^−8^ and 5 × 10^−6^ as genome-wide and suggestive significance threshold to correct false positive findings due to multiple testing (Ji et al., 2019).

### Enrichment analysis of candidate genes

We extended the positions of significant SNPs 150 Kb upstream and downstream and then updated to the Ensembl (UMD3.1 genome version). Identification of the closest genes to significant SNPs was obtained using Ensembl annotation of the UMD3.1 genome version. GO enrichment analysis and Kyoto Encyclopedia of Genes and Genomes (KEGG) enrichment analysis of the candidate genes were performed using the DAVID 6.8 Functional Annotation Tool (https://david.ncifcrf.gov/). In all analyses, the *p*-value < 0.05 was considered significantly different.

Another cost-effective approach to compare, confirm, and locate the most candidate genes related to important traits was to align our results with the QTLdb of UMD3.1, which contains 95,332 QTLs/associations. We identified all the QTLs (<1 Mb) that contained or overlapped with the candidate genes. After matching, the number and function of variants were identified, and these variants were used for subsequent analyses.

## Results

### Phenotypic and estimated genetic parameters

**FIGURE 1:**
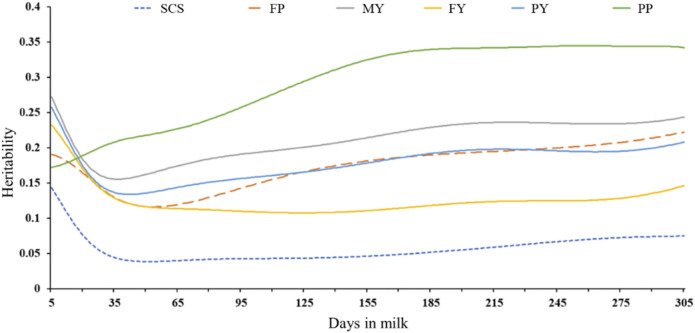
Heritabilities for milk yield, fat yield, protein yield, fat percentage, protein percentage, and SCS estimated with the random regression model for DIM.

Supplementary Figure S1 shows that all the phenotypes follow normal distributions, which can be used for subsequent genome-wide association analysis. The range of inbreeding coefficient for 226,602 animals is 0–0.42. The number of inbred animals is 1,997. Heritabilities for milk yield, fat yield, protein yield, fat percentage, protein percentage, and SCS estimated with the random regression model for DIM are shown in Figure 1. The heritabilities for all phenotypes, except for PP, were reduced from early lactation, were lowest in the peak lactation stage, and increased gradually, remaining quite constant at the mid and late of the lactation stage. Generally, heritabilities for MY, FY, PY, FP, PP, and SCS ranged from 0.16–0.27, 0.11–0.23, 0.13–0.26, 0.12–0.22, 0.17–0.35, and 0.04–0.14 during the lactation, respectively. As expected, heritabilities for SCS are the lowest in all phenotypes.

Genetic correlations between test-day MY, test-day FY, test-day PY, test-day FP, test-day PP, and test-day SCS at different stages of lactation estimated in RRM are ranged from 0.04 to 0.99, 0.04 to 0.99, 0.01 to 0.99, 0.18 to 0.99, 0.33 to 0.99, and -0.02 to 0.99, respectively (Supplementary Table S1). For the six traits, the highest genetic correlation estimates were observed between adjacent test days and the lowest correlations between more distant test days. The genetic correlations for TD5 and TD7, TD50 and TD65, TD140 and TD95, TD125, TD155, TD185, TD215, TD280 and TD245, TD275, TD305 in all traits were larger than 0.95. For SCS, we obtained negative genetic correlations between TD5 and TD215 and TD245. In this study, we emphasized the different physiological stages of the mammary gland across lactation.

### Imputation and quality control

**FIGURE 2:**
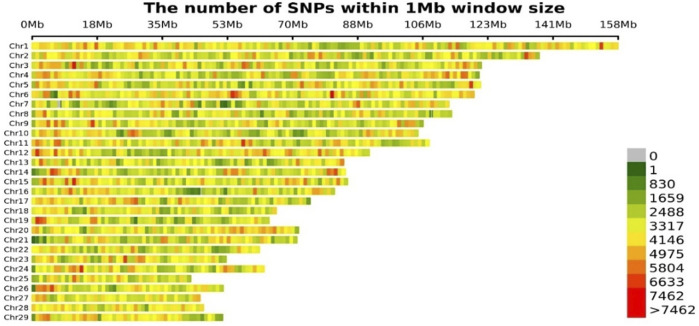
Distribution of SNPs in genome.

The imputation accuracy was 0.95, which was evaluated by the internal information score generated by STITCH itself for the extremely low-coverage whole genome sequencing. The imputation accuracy for GGP Bovine LD v4 and GGP Bovine 50K imputed to GGP Bovine 150K was 0.98 and 0.99, respectively. Then the imputation accuracy for imputed GGP Bovine LD v4 and GGP Bovine 50K to WGS (222 bulls from Run 2 of the 1000 Bull Genome Project as reference panel) was 0.97 and 0.97, respectively. The accuracy for GGP Bovine 150K to WGS was 0.97. All the genotypes imputed to WGS were merged, and the final genotype data file contained 19,105,311 SNPs. After the filtration, 8,686,483 loci and 3,016 individuals were retained to be used in the GWAS. Figure 2 displayed the distribution of SNPs across all autosomes.

### Population stratification assessment

**FIGURE 3:**
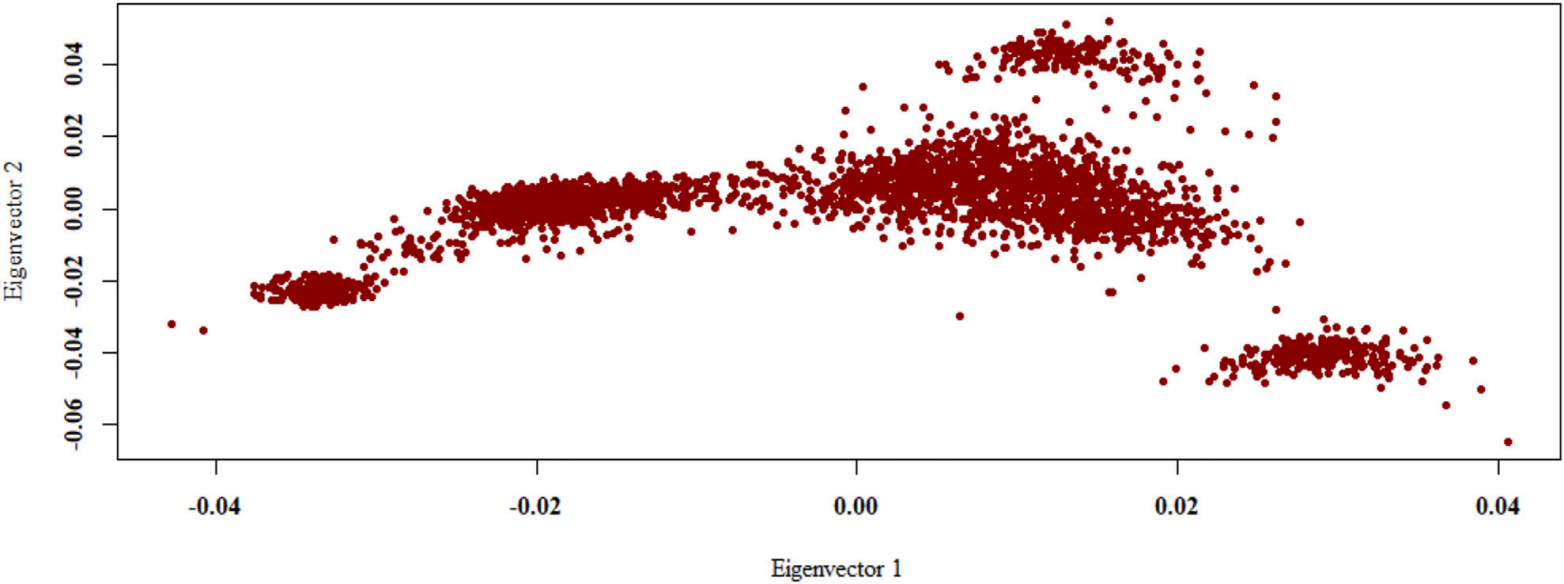
Population structure from the principal component analysis. Population structure is shown as a plot of the first two principal components (PCs). PCA was conducted with the 8,686,483 loci for 3,016 cows.

The PCA revealed that the Shanghai Holstein population are subdivided into five differentiated groups by the first two principal components, which explained 17.25 and 10.96% of the genetic variability in the analysis, respectively, and about 28.21% of the variation is explained by the first three PCs together (Figure 3).

### GWAS results

**FIGURE 4:**
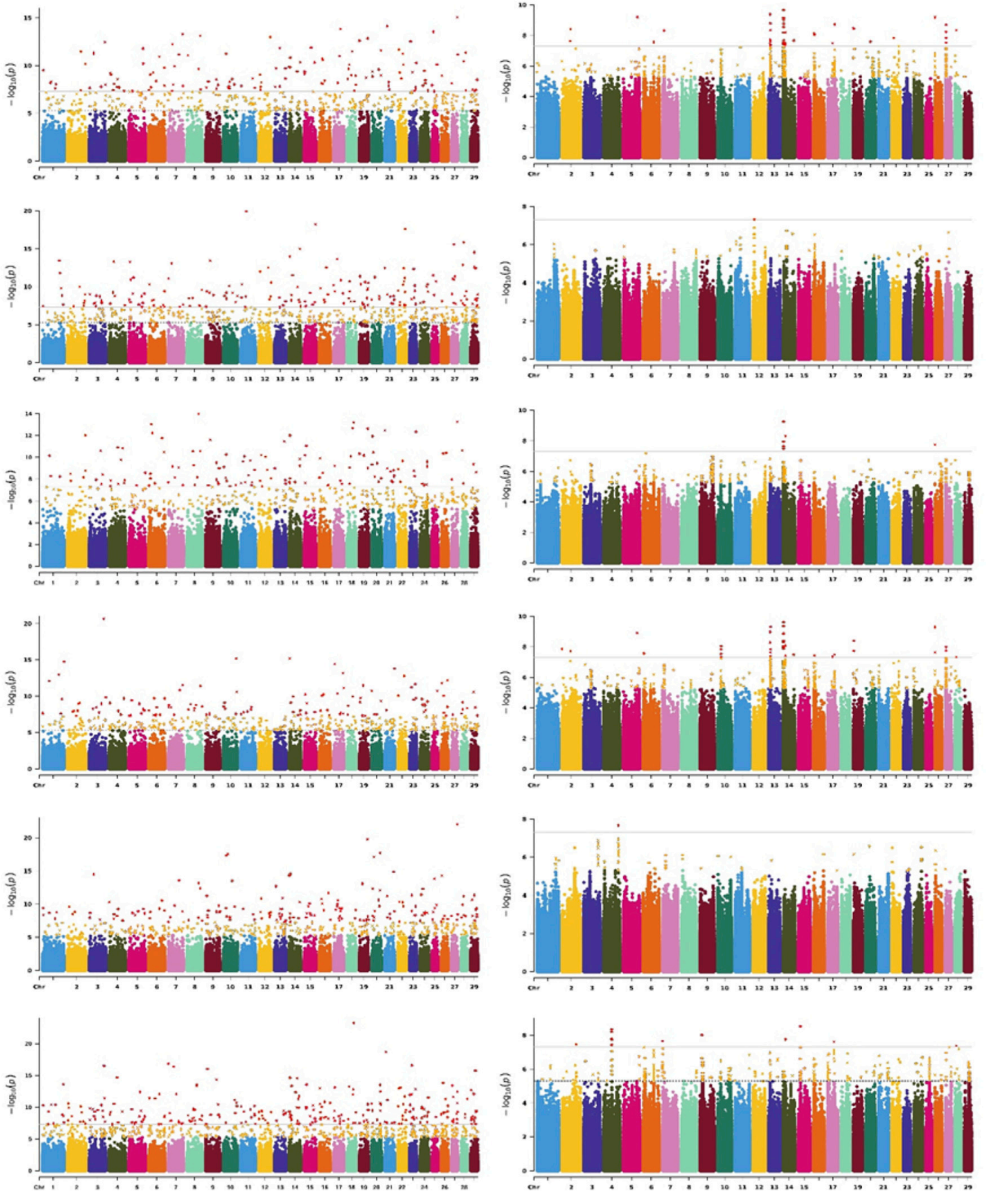
Significance $\left[-\log_{10}\left(Pvalues\right)\right]$ of the association of WGS based on analyses using FarmCPU (left) and MLM (right) with the TD7 of six traits, MY, FP, FY, PP, PY, and SCS (top to down) across 29 autosomes. The grey solid line indicates the Bonferroni multiple test threshold at *p* = 5 × 10^−8^.

Due to the highest genetic correlation estimates being observed between adjacent test days, we only displayed the Manhattan plot of TD7 for all six traits (Figure 4). The Manhattan plot of TD35, TD50, TD140, and TD280 for all six traits are shown in Supplementary Figures S3–S6. The QQ plots are shown in Supplementary Figure S7. The lambda values ranged from 0.921 to 1.042, indicating lower stratification. TD7, TD35, TD50, TD140, and TD280 represented different physiological stages of the mammary gland across lactation (TD7 represented the early, TD50 represented the peak, and TD140 and TD280 represented mid and late lactation, respectively).

We used *p*-value < 5 × 10^−8^ as the threshold, a total of 984, 1,150, 1,291, 1,229, 1,018, and 1,477 significant SNPs detected by FarmCPU for all DIM of MY, FP, FY, PP, PY, and SCS, respectively. 279, 429, 36, 175, 85, and 42 SNPs were identified as significant by MLM for all DIM of MY, FP, FY, PP, PY, and SCS, respectively (Supplementary Table S2 and S3). There are 44, 30, 20, 16, 26, and 41 significant SNPs are both detected by FarmCPU and MLM for all DIM of MY, FP, FY, PP, PY, and SCS, respectively (Supplementary Table S4). We combined the significant SNPs identified by FarmCPU and MLM. Finally, we obtained a total of 1,241, 1,568, 1,316, 1,399, 1,087, and 1,503 significant SNPs (Supplementary Table S5). These findings are consistent with the results of genetic correlation, a large number of the same SNPs were found in mid and late lactation, while the SNPs found in early and peak lactation were mostly specific. The genes that were located within 150 Kb near the significant SNPs were identified as potential candidate genes for the traits investigated. The number of candidate genes identified is listed in Table 2.

**TABLE 2:** Summary statistics for GO and KEGG associated with milk production in Shanghai Holstein population.

Traits	DIM	No. Genes	No. GO	No. KEGG
Milk yield (MY, kg/d)	D7	296	7	0
	D35	339	7	3
	D50	278	11	1
	D140	245	5	1
	D280	179	4	0
Fat yield (FY, kg/d)	D7	232	9	19
	D35	268	8	1
	D50	281	17	7
	D140	336	7	3
	D280	157	11	2
Protein yield (PY, kg/d)	D7	438	6	5
	D35	266	5	7
	D50	280	5	5
	D140	173	3	2
	D280	122	6	5
Fat (FP, %)	D7	450	15	16
	D35	263	18	8
	D50	290	7	2
	D140	308	13	1
	D280	311	3	0
Protein (PP, %)	D7	268	11	11
	D35	208	17	2
	D50	303	10	2
	D140	268	11	17
	D280	325	30	5
SCS	D7	559	7	6
	D35	389	5	11
	D50	373	13	4
	D140	180	15	1
	D280	283	3	5

We paid more attention to the candidate genes which contained or were near to the most significant SNPs associated with different milk production in five lactation stages (TD7, TD35, TD50, TD140, and TD280). For MY, the candidate genes contained the most significant SNPs for TD7, TD35, TD50, TD140, and TD280 were *GRM4*, *VEPH1*, *SCRIB*, *PLBD1,* and *LAMA3*, respectively. For FP, the candidate genes contained the most significant SNPs for TD7, TD35, TD50, TD140, and TD280 were *ATP2B2*, *NRP1*, *BOP1*, *DGAT1,* and *DGAT1*, respectively. The most significant SNP associated with FP at early lactation was BTA22:55263235 (*p*-value = 2.37E-18). The most significant SNPs for FP at mid and late lactation both were BTA14:1801116 (for TD140: *p*-value = 6.96E-56; for TD280:*p*-value = 7.47E-59). For FY, the candidate genes contained the most significant SNPs for TD7, TD35, TD50, TD140, and TD280 were *DSP, MAML3, PRKG1, WDR34,* and *SLC1A3,* respectively. For PP, the candidate genes contained the most significant SNPs for TD7, TD35, TD50, TD140, and TD280 were *DCLK2, AHCTF1, OCLN, MROH1,* and *HSF1*, respectively. The most significant SNP associated with PP at late lactation was BTA14:1807140 (*p*-value = 1.26E-17). For PY, the candidate genes contained the most significant SNPs for TD7, TD35, TD50, TD140, and TD280 were *CTNND2*, *CSMD3*, *WWOX*, *ARHGAP10,* and *LMAN2L*, respectively. For SCS, the candidate genes contained the most significant SNPs for TD7, TD35, TD50, TD140, and TD280 were *NFKBIE*, *ABCF1*, *MYZAP*, *TTLL7,* and *DNAH9*, respectively.

We further identified the genes which were candidate genes for more than two lactation stages or traits. For MY, there were 18 candidate genes for at least two lactation stages, including *NDST4*, *ICAM2*, *KCNMA1*, *LRP5*, *KALRN*, *IQCA1*, *MANBA*, *SCRIB*, *COL22A1*, *MORN1*, *APBA2*, *ZMYND8*, *WWOX*, *BFAR*, *CECR2*, *GALNT16*, *SPOP,* and *CPEB3*. For FP, a total of 20 candidate genes for at least two lactation stages contained significant SNPs, including *DGAT1, ADAMTS3, ZKSCAN7, CTNNA3, CDH23, ELM O 1, SLC15A5, ESR1, NRP1, BOP1, RPH3A, ATRNL1, FAM21A, MGST1, USH2A, WDR87, SYNRG, RANBP17, ANKRD55,* and *PRIM2*. For FY, 11 genes associated with at least two lactation stages. For PP, 18 genes involved in at least two lactation stages, including *ZMYND8, AHCTF1, TSHR, RALYL, RYR2, ORC2, MAP1S, MT O 1, NRP1, TECPR2, LRP5, NADSYN1, SMC5, KCNQ5, MAP2K6, OCLN, PBX1,* and *PRKG1*. For PY, 15 candidate genes involved in at least two lactation stages, including *WWOX, TMEM132C, NDST4, GUCY1A2, CTNND2, MANBA, MCC, KCNIP1, ITGA2, CTNNA3, SCRIB, CCDC33, MACROD2, PITPNB,* and *FDXR*. For SCS, 27 candidate genes involved in at least two lactation stages, including *PCDH15, ELM O 1, LDB2, SH3GL2, COL22A1, NUDCD1, HMCN1, CCDC63, GALNS, ADTRP, C1QTNF7, LPAR1, MYZAP, PLCB1, SLC38A9, LANCL2, SLC35F3, DKK2, KCNIP4, TRIM11, RERG, ACOXL, DDX54, DNAH9, ERICH1, MTA1,* and *B3GALNT2*.

### Functional annotation of candidate genes

The *p*-value adjusted using the Bonferroni approach (*p*-value < 0.05) was considered to be the threshold value for significantly enriched GO terms and pathways. As shown in Table 2, the number of GO terms and KEGG pathways were significantly enriched for six milk production traits across lactation in Shanghai Holstein. In the current study, gene set enrichment analyses revealed that several terms, such as response to external stimulus (GO:0048870), detection of stimulus (GO:0051606), negative regulation of response to stimulus (GO:0048585), and development process were found for almost all traits in almost all lactation stages (Supplementary Table S6–S11). It is interesting that feeding behavior (GO:0007631) was identified for milk yield. For FP, the GO term analysis identified the immune effector process (GO:0002252) and immune response (GO:0003823) in peak lactation. In addition, sexual reproduction (GO:0019953) and reproductive process (GO:0022414) were identified in mid lactation (Supplementary Table S6). For MY, the GO terms were most involved in the biological process and cellular component, such as intracellular (GO:0005622), regulation of signaling (GO:0023051), and plasma membrane part (GO:0044459) (Supplementary Table S8). For PP, several GO terms related to the development and growth process were identified several in peak and late lactation. 2 GO terms related to reproduction were identified in late lactation (Supplementary Table S9). For SCS, response to chemical (GO:0042221) was identified in peak and mid lactation. In late lactation, the GO terms were related to growth (Supplementary Table S11). The pathways significantly enriched are listed in Supplementary Table S12–S17, of which several pathways were implicated in signal transduction, including the MAPK signaling pathway (bta04010), Rap1 signaling pathway (bta04015), Ras signaling pathway (bta04014), chemokine signaling pathway (bta04062), Jak-STAT signaling pathway (bta04630), oxytocin signaling pathway (bta04921), and sphingolipid signaling pathway (bta04071); one pathway, olfactory transduction (bta04740), was identified in PP in early lactation and SCS in peak and mid lactation and MY in mid lactation. One pathway was associated with PY, namely, inflammatory mediator regulation of TRP channels (bta04750).

The number and function of variants identified using QTL annotation are listed in Table 3. The significant SNPs associated with MY in late lactation and PP in mid and late lactation were mainly overlapped with milk-related and production-related QTL regions. The SNPs were identified variants and were used for subsequent analyses.

**TABLE 3:** Number of significant SNPs for QTL annotation with different DIM of milk production.

Traits	DIM	Exterior	Health	Milk	Production	Reproduction
Milk yield (MY, kg/d)	D7	23	25	54	60	38
	D35	27	16	60	71	43
	D50	28	28	61	71	28
	D140	49	54	122	116	80
	D280	166	172	298	244	178
Fat yield (FY, kg/d)	D7	24	18	57	76	39
	D35	26	28	61	75	37
	D50	34	23	66	69	48
	D140	18	32	60	63	40
	D280	17	12	34	43	28
Protein yield (PY, kg/d)	D7	38	41	86	91	57
	D35	15	23	66	57	37
	D50	22	21	54	59	33
	D140	19	19	35	41	20
	D280	11	9	23	26	19
Fat (FP, %)	D7	30	45	80	88	46
	D35	217	238	385	327	231
	D50	256	351	534	505	275
	D140	261	401	590	591	293
	D280	265	435	649	664	297
Protein (PP, %)	D7	23	23	70	65	38
	D35	19	13	38	40	26
	D50	17	24	53	53	36
	D140	147	171	316	252	177
	D280	151	173	333	274	184
SCS	D7	59	31	115	137	82
	D35	32	42	87	94	58
	D50	35	27	85	73	51
	D140	24	18	57	50	33
	D280	13	17	39	46	26

## Discussion

In this research, we estimated various genetic parameters in a large population of Shanghai Holstein that had been regularly measured for six major dairy traits throughout lactation since 1995. This estimation was performed by using a random regression model for the first time in Shanghai. Currently, there are many studies for different Holstein populations (Buaban et al., 2021; Salimiyekta et al., 2021; Fathoni et al., 2022; Sungkhapreecha et al., 2022). We found that the genetic correlation between different test days for milk production was less than one, implying that the different test days had a different additive genetic variance. Oliveira H. et al. (2019) demonstrated that distinct genomic regions affect milk production traits across test days in a whole lactation (Oliveira H. R. et al., 2019). Compared with the genetic correlation estimated in this study, the genetic correlations between TD5 and TD7, TD95 to TD185, and TD245 to TD305 were all extremely high. This means that genetic improvement of one test day of milk production traits could result in a correlated response in the correlated traits. Although there have been many GWAS analyses of milk production traits, elucidating the molecular mechanisms of these traits in other populations can provide new insights into understanding the genetic basis of these traits in dairy cows. Our study subdivided milk production traits during lactation and, more precisely, found significant SNPs that affected different test days.

Currently, there are many studies on the submodels in the random regression test day model. The results of these studies showed that the lactation curves of milk production traits obtained by different researchers were also quite different (El Faro et al., 2008; Zhou and Zhang, 2021; Paiva et al., 2022). Since 1994, with the application of Legendre polynomials in the random regression test day model, research on its order has continued. Li J. et al. (2020) found that for local Chinese Holstein populations, models with third-, fourth-, and fifth-order of Legendre polynomials (LP) led to similar estimates of genetic parameters and predictive ability. Models with higher order obtained lower Akaike information criterion (AIC) and Bayesian information criterion (BIC) values, which was in line with previous studies (Pereira et al., 2013). This means models with LP5 fit data best regardless of complexity. Costa et al. (2008) used fifth-order Legendre polynomial to fit two random effects. Also, RRM based on Legendre polynomials is sensitive to too few records per cow, especially for estimating extreme values of the lactation curve. At the same time, to avoid non-convergence in the RRM due to too few records per cow, we eliminated individuals with fewer than three records when filtering the data.

In our study, except for FP, other traits showed that heritability reached its maximum in early lactation. The heritability of MY varied from 0.16 to 0.27, with the lowest value in peak lactation. In general, the trend for MY heritabilities was like the trend found by Kheirabadi (2019) and Jamrozik and Schaeffer (2012). Kheirabadi (2019) reported that the heritabilities of MY increased with stage of lactation from 0.05 to 0.09 for DIM 5 to 0.24 to 0.25 for DIM 305 for the Iran Holstein population. Jamrozik and Schaeffer (2012) reported that the heritabilities expressed daily were relatively uniform across DIM, except for DIM ranging from 5 to 25. Several studies have reported that the heritabilities of MY in early and late lactation were larger than the value in peak lactation, which is consistent with our results. SCS can reflect the health of the mammary glands, but the low heritability of SCS is an important factor limiting mastitis-resistant breeding. In our research, the heritabilities for DIM ranged from 0.04 (TD51) to 0.14 (TD5). Jamrozik and Schaeffer (2012) found that SCS reached a maximum value in the early lactation, then gradually decreased, and reached a minimum at the peak lactation, then increased steadily and slowly across the lactation. Zakizadeh and Jafari (2014) reported that the heritabilities varied from 0.04 (in early lactation) to 0.136 (in late lactation) for SCS.

We analyzed the genetic correlation between different test days and found that it was highest (close to 1) on adjacent test days but gradually decreased with increasing DIM intervals, which was consistent with previous studies. Jakobsen et al. (2002a) found a genetic correlation between different test days greater than 0.4 (Jakobsen et al., 2002b). Elahi Torshizi et al. (2016) reported that the genetic correlation between different test days varied from 0.47 to 0.98 (Elahi Torshizi, 2016). There was a significant negative genetic correlation between milk production traits in early and late lactation. These negative genetic correlations may be due to difficulties in modeling milk production traits in early lactation when cows are experiencing postpartum stress and lack of energy. Soumri et al. (2020) found the genetic correlation for SCS between test days from -0.11 to 0.99 by using fifth-order Legendre polynomial to fit random effects, which is like the findings in our research (-0.02 to 0.99 across the whole lactation for SCS) (Soumri et al., 2020). The genetic correlation for different DIM is not 1, which means that the additive genetic variance in different DIM is different, which also means that the RRM is used to analyze longitudinal data (e.g., milk production traits). Also, the extremely high genetic correlation between TD95 to TD185 and TD245 to TD305 can explain why the measurement and recording of milk production traits during some test days can be simplified without compromising the reliability of parameter estimates using the RRM.

SNP chips are customized chips based on existing SNP information, and new SNP cannot be found. The coverage of genotyping-by-sequencing accounts for only about 5% of the whole genome and many SNPs are missed. WGS can find SNPs on a genome-wide scale without causing the omission of SNPs (Ye et al., 2018). Compared with SNP chips and GBS, GWAS based on WGS has significant advantages, including that WGS is based on the entire genome to scan and detect SNPs, and the mapping is more accurate (Wu et al., 2019). Therefore, the use of WGS data is expected to improve the detection of QTL, such as the GWAS by using 234 bulls’ WGS data in the 1000 Bull Genomes Project (Daetwyler et al., 2014). Although the cost of WGS has decreased, sequencing a large number of individuals for WGS data is still exorbitant. With the development of genotype imputation software, a low-cost method to increase the number of animals with WGS data has been proposed by imputing the lower-density microarray data to the WGS level. Recently, GWAS using imputed WGS data has been widely used in different livestock, such as pigs (Li X. et al., 2020), chickens (Ni et al., 2017; Visscher et al., 2017), cattle (Van Binsbergen et al., 2015; Zhang et al., 2016), and horses (Asadollahpour Nanaei et al., 2020). Especially for cattle, many studies have detected significant important candidate genes by using imputed WGS data in GWAS (Chen N. et al., 2018). In our research, we imputed low- and medium-density SNP chips and GGRS by using a high-coverage WGS-based imputation reference panel (222 bulls from Run 2 of the 1000 Bull Genome project) to WGS data, which is consistent with imputation strategies used in other studies. It has been shown that the use of imputed WGS data in cattle is effective in detecting significant SNPs peaks that were not previously found when using high-density SNP chips in GWAS (Yoshida and Yáñez, 2022). Simultaneously, some authors detected significant SNPs in almost all autosomes by using the imputed WGS data to conduct GWAS on milk production traits, which is in line with our results. In this study, these SNPs identified on different DIM partially overlapped (Sanchez et al., 2017). At the same time, we used a very strict significance threshold (Bonferroni correction treats all variants as independent) that may reduce detection power but minimizes the risk of false positive QTLs.

The genes found in at least two lactation stages or traits and contained or near the most significant SNPs associated with milk production traits were the most important candidate genes in our study. For all six traits studied, there are many common candidate genes detected in TD35 and TD50, such as seven genes among 20 candidate genes for FP, which may be due to the relatively close lactation interval of TD35 and TD50, and the high genetic correlation (greater than 0.9); thus, the mechanisms affecting the traits are similar. *NDST4* is associated with milk fever in the U.S. Holstein cattle (Cavani et al., 2022). In a previous study of milk production traits in Canadian Holstein at different lactation stages, *SCRIB* on BTA14 was a candidate gene for MY and was associated with TD95 to TD215 of PY (Oliveira et al., 2018). Jiang et al. (2010) found that *COL22A1* was an important candidate gene for MY, FP, and PY by conducting GWAS in Chinese Holstein cattle (Jiang et al., 2010). *DGAT1* was detected in the mid and late lactation of FP, which mainly had positive effects on FY and negative effects on MY and PY. Studies have reported that *MGST1* and *SLC15A5* are associated with FY (Jiang et al., 2019). *ADAMTS3* was detected in early, mid, and late lactation, and *ADAMTS3* has been reported to be associated with MY and PY. It is worth noting that *ADAMTS3* is also significantly associated with the longevity of cows (Mészáros et al., 2014). *TECPR2* is related to the heat resistance traits of Chinese cattle, and SNPs located in the gene can be used as molecular markers for Chinese cattle breeding (Ma et al., 2021). Also, *TECPR2* was found to be a candidate gene for SCS in Thai Holstein cattle (Buaban et al., 2022). *PRKG1* plays a key role in lipolysis and is an important candidate gene for fatty acids in milk (Shi et al., 2019, 1). Meanwhile, *PRKG1* was associated with tick resistance in cattle. Our study further supports the importance of this gene in disease resistance traits (Alshawi et al., 2019). *TMEM132C, CTNND2*, and *PCDH15* have been found to be associated with milk production traits (Yodklaew et al., 2017, 2017; Gan et al., 2020). *HMCN1* is known to be associated with age-related macular degeneration, and polymorphisms within the *HMCN1* gene are associated with diabetes in humans (Fisher et al., 2007). This reflects a consistent increase in SCS with age and the progression of lactation, which is consistent with the findings of this study. *DKK2* is involved in adipocyte lipogenesis, which may play a role in fat secretion in milk (Li et al., 2010). *ACOXL* is associated with lipid metabolism and glucose pathways (Klein et al., 2020). *PLCB1* plays multiple biological roles in human diseases, such as inflammation, cell proliferation, and schizophrenia. *DNAH9* affects milk’s volatile fatty acid content (Nakamura et al., 2018). *B3GALNT2* was found in a GWAS study of milk production traits in Danish Jersey and Holstein cattle by Poulsen et al. (Buitenhuis et al., 2014). A previous study showed that *ATP2B2* is associated with milk production traits and mastitis (Ogorevc et al., 2009), and the most significant SNP (BTA22:55263235, *p*-value = 2.37E-18) in the GWAS of TD7 FP is located in the intronic region of *ATP2B2*. *PLBD1* is an important candidate gene for fatty acid composition in milk (Atashi et al., 2020). The most significant SNP for PP of TD280 was BTA14:1807140 with a *p*-value = 1.26E-17, which was located on *HSF1*, and *HSF1* plays a crucial role in heat stress response. A previous study found an SNP in the 3′-UTR (g.4693G>T) of *HSF1* that was related to thermo tolerance in Chinese Holstein cattle through association analysis (Li et al., 2011). *NFKBIE* may control the response to several bacterial and viral pathogens and vaccine responses (Lundbo et al., 2016).

Only a few studies have focused on time-dependent genetic associations in livestock to date, but the investigation of the association at certain lactation stages seems to be a promising approach to detect loci associated with milk production (Strucken et al., 2012). Thus, we analyzed how the genetic influence of genomic regions changes during the most critical stages of lactation in our study. We found that the genetic influence on milk production traits varies throughout lactation, which is crucial to enable more efficient genetic selection for these traits and for better management practices, especially for farms or breeders to select high-yielding or milk long-lasting dairy cows. Milk production is related to the stage of lactation, including early lactation, peak lactation, mid lactation, and late lactation. Early lactation is known to be a critical period, especially in high-yielding dairy cows (Deng et al., 2019). Selecting for maximum milk production during lactation early in lactation would improve persistency by lowering the rate of decrease after peak yield (Ferris et al., 1985). Peak milk yield plays a decisive role during the whole lactation period. Zhang et al. (2019) reported that for every 1 kg increase in peak milk production, the yield per primiparous cow increases by about 400 kg. The effect of heat stress on milk yield has been shown to be highest in mid or late lactation. Different genes may be involved in handling different disturbances, explaining the genetic difference among the milk production traits in different lactation stages (Poppe et al., 2021). Candidate genes were only detected at the beginning of lactation showed that the impact on milk production traits must be diminishing in late lactation and suggested that these genes are associated with lactogenesis at the onset of lactation. Candidate genes were detected for all stages of lactation, which could therefore play a role in the immune response of the mammary gland and prevents inflammation during lactation (Strucken et al., 2012). We can use a genomic selection model that combines with markers (significantly associated with different stages of lactation) fit as fixed effects selected from the results of a GWAS (Yin et al., 2020). For example, *MROH1*, an important candidate gene for milk protein composition, is located in a 1.85–2.11 Mb region on BTA14 that has been shown to be associated with 305-days and peak milk production in cows. In addition, the model for selecting is also important. RRM is a feasible alternative to yield more accurate selection and culling decisions. RRM provides information about the temporal variation of biological processes underlying the studied traits to exploit for management and breeding purposes (Oliveira H. et al., 2019).

## Conclusion

In our study, an RRM with fifth-order of Legendre polynomials was an appropriate model for genetic evaluation of six milk production traits in Shanghai Holstein populations. The main results showed that genetic parameters and breeding values were successfully estimated. The results of genetic correlations demonstrated that combining the milk production traits tested on different lactation into a single trait can lead to inaccurate estimates of the genetic value of dairy cows. At the same time, the measurement and recording of milk for some adjacent lactation periods can be simplified without affecting the reliability of parameter estimation using RRM. Then, we detected significant SNPs and candidate genes associated with different traits in different lactation stages, mainly including milk-related genes (*DGAT1, MGST1, PTK2, SCRIB, PRKG1, CTNND2, MROH1, ATP2B2,* and *DNAH9*), disease-related genes (*LY6K, COL22A1, TECPR2, KALRN, CYP7B1, HMCN1,* and *PLCB1*), heat stress–related genes (*ITGA9, NDST4, TECPR2,* and *HSF1*), and reproduction-related genes (*7SK* and *DOCK2*). The genes and QTLs related to heat stress are important to investigate the mechanism of response to heat stress, such as *ITGA9*, which can act as an important gene for heat-resistant breeding of Shanghai Holstein.

## Data Availability

The data presented in the study are deposited in the Alphaindex repository (http://alphaindex.zju.edu.cn/alphaindex/index.php).
