# Corrigendum: Genetic parameters and genome-wide association for milk production traits and somatic cell score in different lactation stages of Shanghai Holstein population

**DOI:** 10.3389/fgene.2025.1604082

**Published:** 2025-04-11

**Authors:** Dengying Liu, Zhong Xu, Wei Zhao, Shiyi Wang, Tuowu Li, Kai Zhu, Guanglei Liu, Xiaoduo Zhao, Qishan Wang, Yuchun Pan, Peipei Ma

**Affiliations:** ^1^ Shanghai Key Laboratory of Veterinary Biotechnology, Department of Animal Science, School of Agriculture and Biology, Shanghai Jiao Tong University, Shanghai, China; ^2^ Hubei Key Laboratory of Animal Embryo and Molecular Breeding, Institute of Animal Husbandry and Veterinary, Hubei Provincial Academy of Agricultural Sciences, Wuhan, China; ^3^ Shanghai Dairy Cattle Breeding Centre Co, Ltd, Shanghai, China; ^4^ Department of Animal Breeding and Reproduction, College of Animal Science, Zhejiang University, Hangzhou, China

**Keywords:** Shanghai Holstein population, milk production traits, genetic parameter, genome-wide association study, different stages of lactation

In the published article, there was an error in the **Funding** statement. There was one important grant missing (the Natural Science Foundation of Shanghai (22ZR1431500)). The correct Funding statement appears below.

