# Supplementary material for: Genetic parameters and genome-wide association for milk production traits and somatic cell score in different lactation stages of Shanghai Holstein population
Source: Front Genet. 2022 Sep 5;13:940650. doi: 10.3389/fgene.2022.940650 (PMC9483179; doi:10.3389/fgene.2022.940650)
Supplement: Supplementary file 1 [file DataSheet1.docx]

**Figure S1.** Normal distribution of six milk production and quality traits of the Shanghai Holstein population.


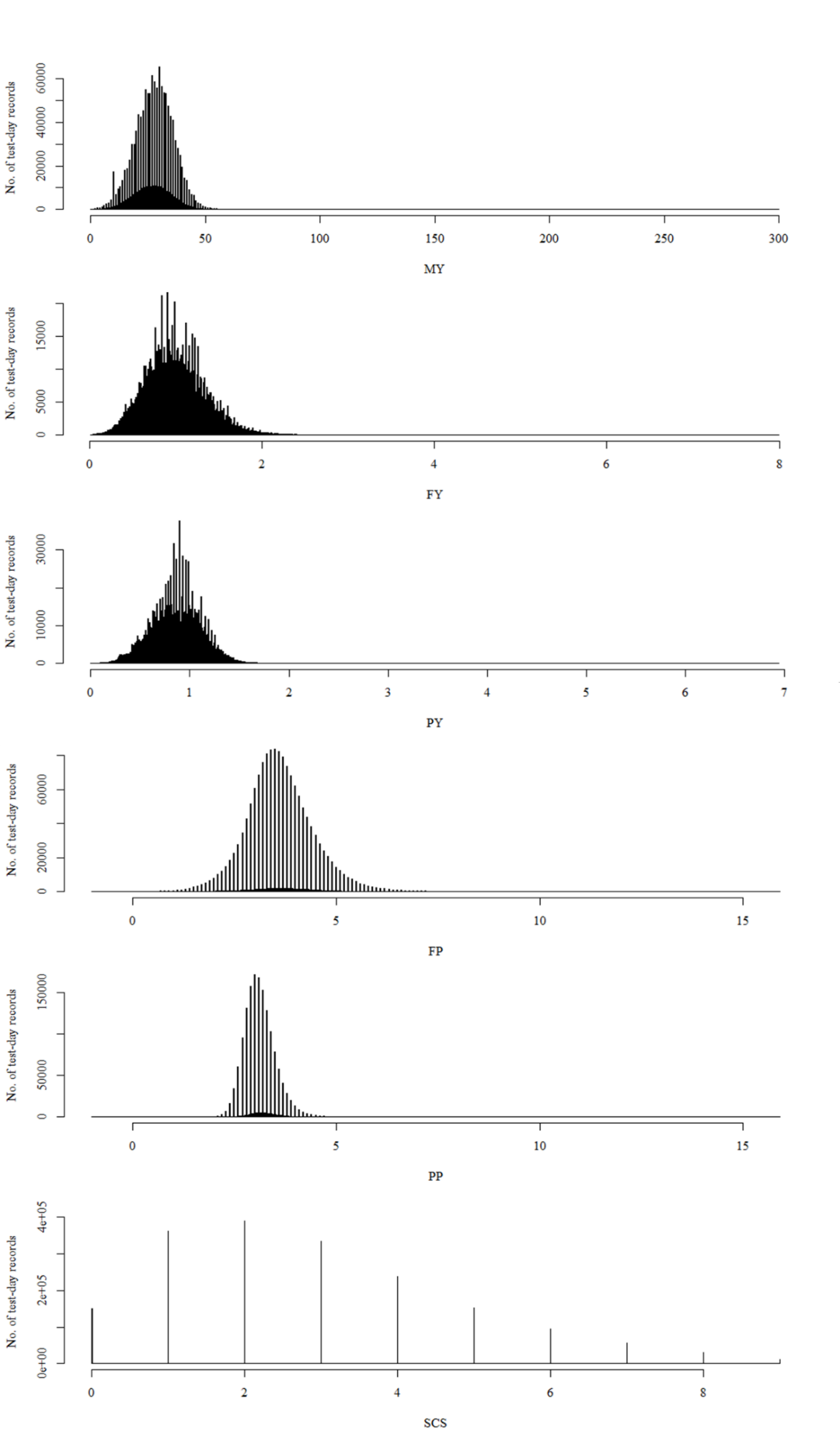


**Figure S2.** Significance $\left[ -\log_{10} (Pvalues) \right]$of the association of WGS based on analyses using FarmCPU (left) and MLM (right) with the TD35 of 6 traits, MY, FP, FY, PP, PY and SCS (top to down) across 29 autosomes. The grey solid line indicates the Bonferroni multiple test threshold at *p* = 5×10^-8^.


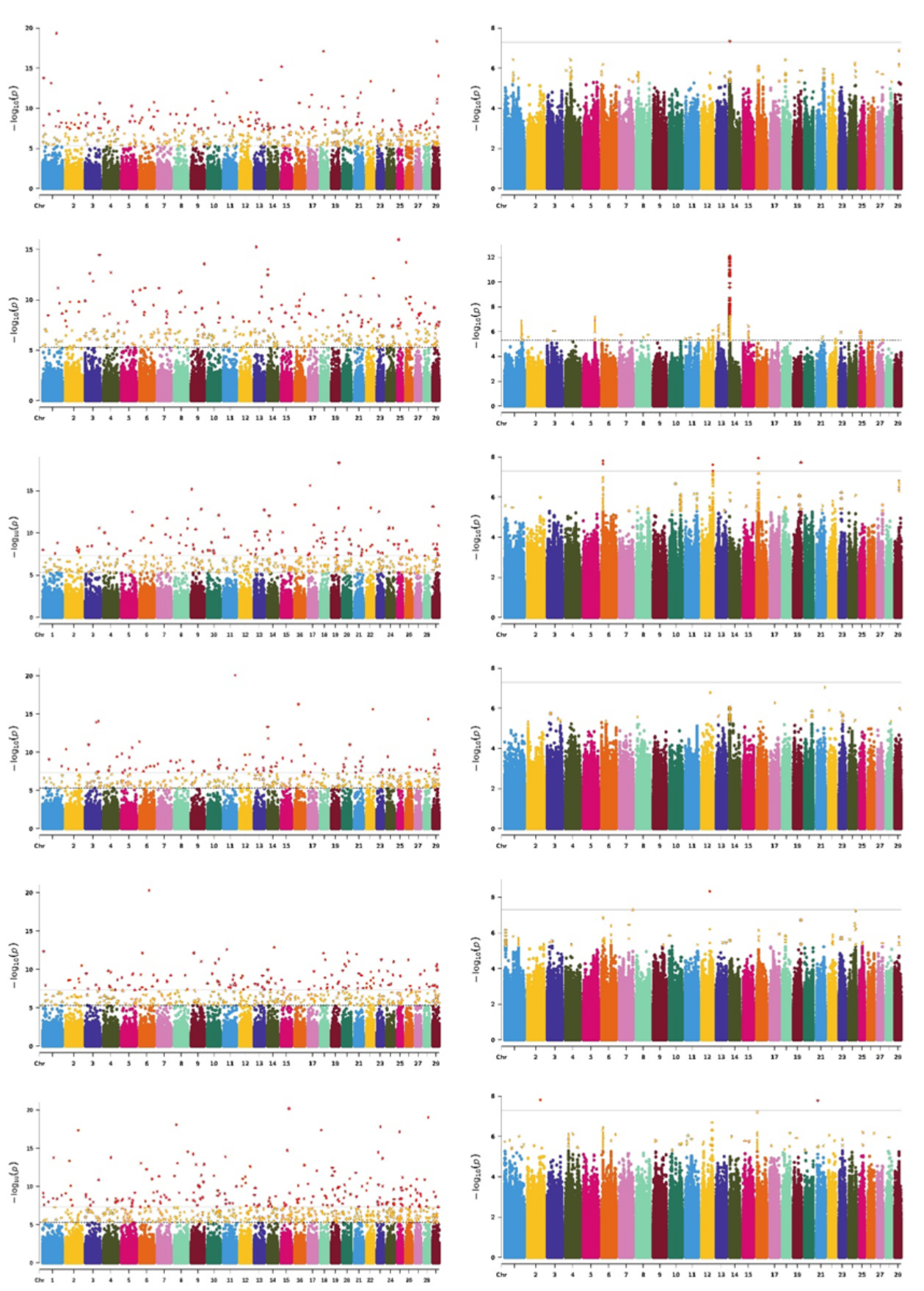


**Figure S3.** Significance $\left[ -\log_{10} (Pvalues) \right]$of the association of WGS based on analyses using FarmCPU (left) and MLM (right) with the TD50 of 6 traits, MY, FP, FY, PP, PY and SCS (top to down) across 29 autosomes. The grey solid line indicates the Bonferroni multiple test threshold at *p* = 5×10^-8^.


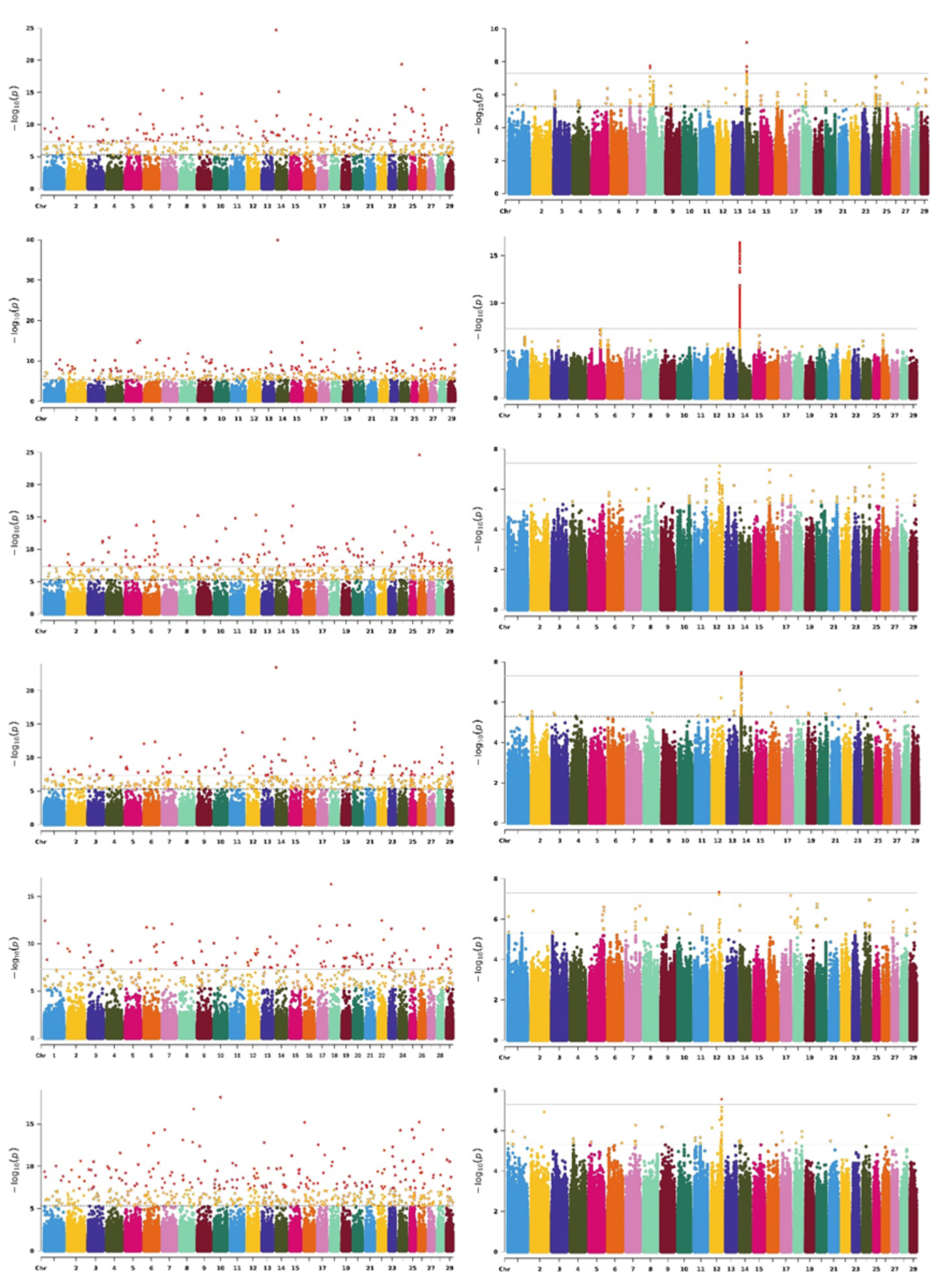


**Figure S4.** Significance $\left[ -\log_{10} (Pvalues) \right]$of the association of WGS based on analyses using FarmCPU (left) and MLM (right) with the TD140 of 6 traits, MY, FP, FY, PP, PY and SCS (top to down) across 29 autosomes. The grey solid line indicates the Bonferroni multiple test threshold at *p* = 5×10^-8^.


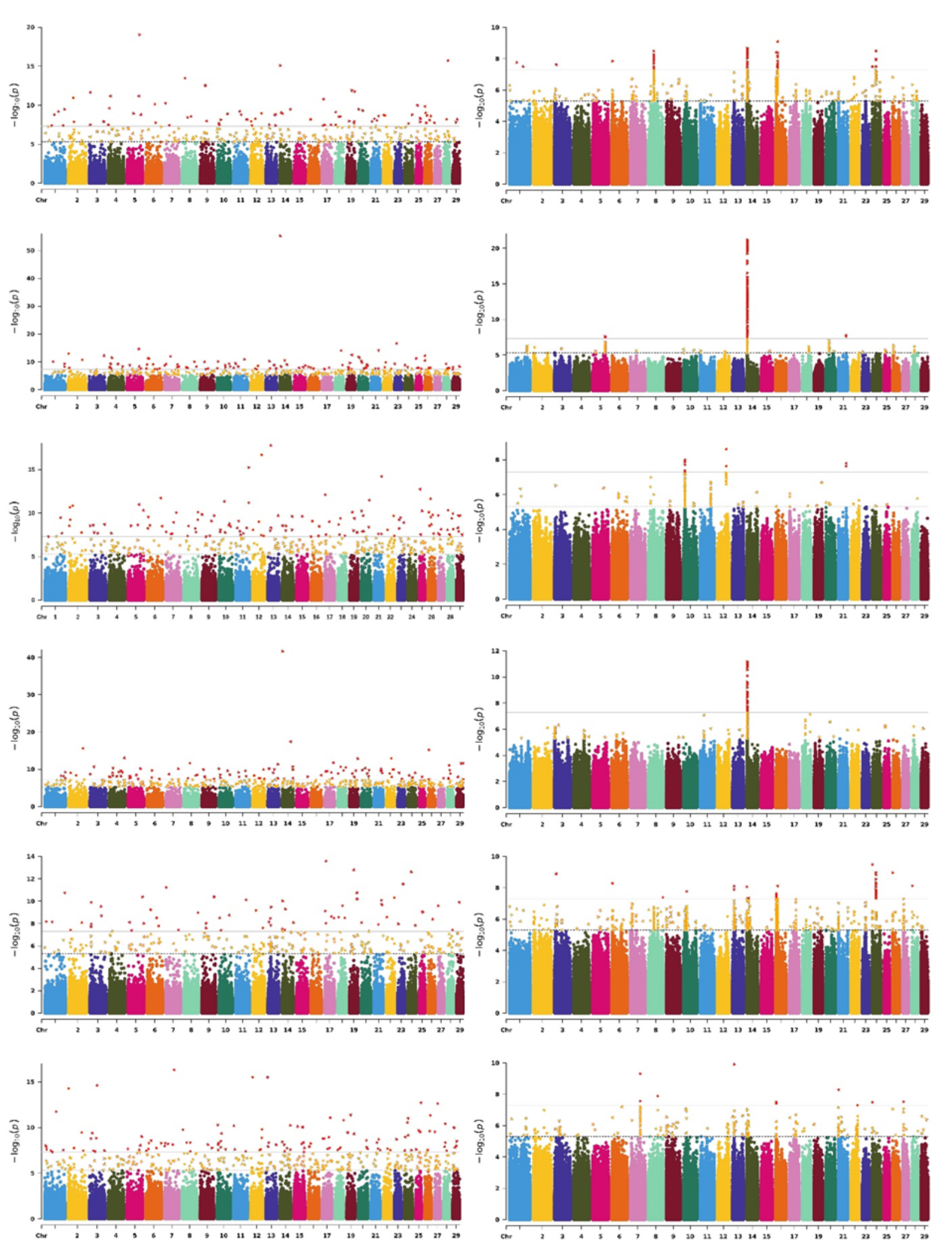


**Figure S5.** Significance $\left[ -\log_{10} (Pvalues) \right]$of the association of WGS based on analyses using FarmCPU (left) and MLM (right) with the TD280 of 6 traits, MY, FP, FY, PP, PY and SCS (top to down) across 29 autosomes. The grey solid line indicates the Bonferroni multiple test threshold at *p* = 5×10^-8^.


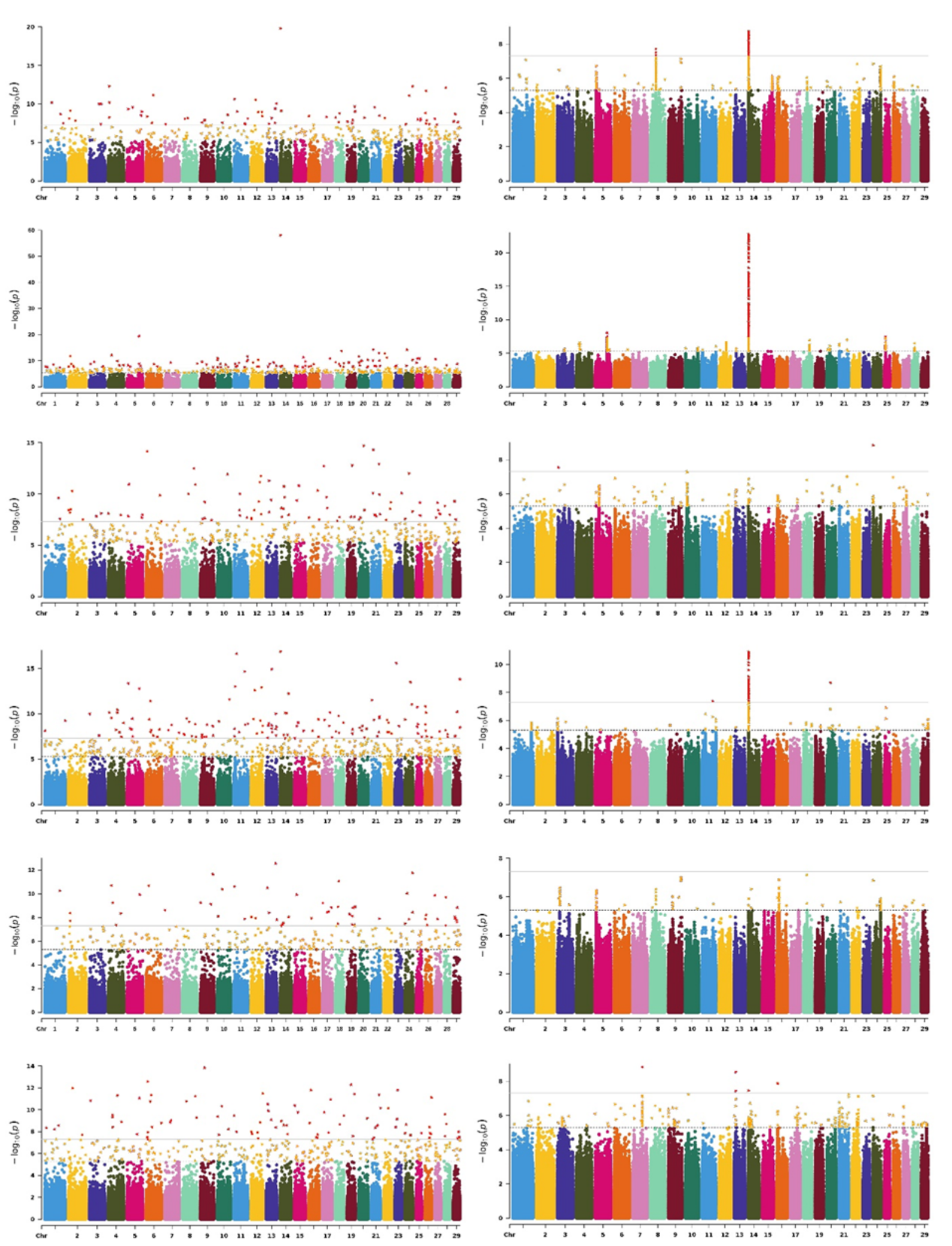


**Table S1.** Genetic correlation of different DIM milk production and quality traits

| **Traits** | **DIM** | **5** | **7** | **35** | **50** | **65** | **95** | **125** | **140** | **155** | **185** | **215** | **245** | **275** | **280** | **305** |
| --- | --- | --- | --- | --- | --- | --- | --- | --- | --- | --- | --- | --- | --- | --- | --- | --- |
| MY | 5 | 1.00 | 1.00 | 0.55 | 0.30 | 0.16 | 0.06 | 0.05 | 0.05 | 0.05 | 0.04 | 0.05 | 0.10 | 0.18 | 0.20 | 0.23 |
|  | 7 |  | 1.00 | 0.60 | 0.35 | 0.21 | 0.11 | 0.09 | 0.09 | 0.09 | 0.08 | 0.09 | 0.13 | 0.21 | 0.22 | 0.25 |
|  | 35 |  |  | 1.00 | 0.96 | 0.89 | 0.79 | 0.72 | 0.69 | 0.67 | 0.63 | 0.61 | 0.58 | 0.53 | 0.52 | 0.47 |
|  | 50 |  |  |  | 1.00 | 0.98 | 0.92 | 0.85 | 0.82 | 0.79 | 0.76 | 0.72 | 0.68 | 0.60 | 0.58 | 0.51 |
|  | 65 |  |  |  |  | 1.00 | 0.97 | 0.92 | 0.89 | 0.86 | 0.82 | 0.79 | 0.73 | 0.64 | 0.63 | 0.55 |
|  | 95 |  |  |  |  |  | 1.00 | 0.98 | 0.97 | 0.95 | 0.91 | 0.87 | 0.82 | 0.74 | 0.72 | 0.65 |
|  | 125 |  |  |  |  |  |  | 1.00 | 1.00 | 0.99 | 0.96 | 0.93 | 0.89 | 0.82 | 0.80 | 0.74 |
|  | 140 |  |  |  |  |  |  |  | 1.00 | 1.00 | 0.98 | 0.95 | 0.91 | 0.85 | 0.84 | 0.78 |
|  | 155 |  |  |  |  |  |  |  |  | 1.00 | 0.99 | 0.97 | 0.94 | 0.88 | 0.86 | 0.80 |
|  | 185 |  |  |  |  |  |  |  |  |  | 1.00 | 0.99 | 0.97 | 0.92 | 0.90 | 0.84 |
|  | 215 |  |  |  |  |  |  |  |  |  |  | 1.00 | 0.99 | 0.95 | 0.94 | 0.88 |
|  | 245 |  |  |  |  |  |  |  |  |  |  |  | 1.00 | 0.98 | 0.97 | 0.93 |
|  | 275 |  |  |  |  |  |  |  |  |  |  |  |  | 1.00 | 1.00 | 0.98 |
|  | 280 |  |  |  |  |  |  |  |  |  |  |  |  |  | 1.00 | 0.99 |
|  | 305 |  |  |  |  |  |  |  |  |  |  |  |  |  |  | 1.00 |
| FP | 5 | 1.00 | 1.00 | 0.73 | 0.46 | 0.29 | 0.18 | 0.19 | 0.20 | 0.21 | 0.20 | 0.18 | 0.19 | 0.23 | 0.24 | 0.28 |
|  | 7 |  | 1.00 | 0.75 | 0.50 | 0.32 | 0.20 | 0.21 | 0.22 | 0.23 | 0.22 | 0.21 | 0.21 | 0.25 | 0.26 | 0.30 |
|  | 35 |  |  | 1.00 | 0.94 | 0.83 | 0.68 | 0.62 | 0.61 | 0.61 | 0.62 | 0.62 | 0.61 | 0.59 | 0.58 | 0.57 |
|  | 50 |  |  |  | 1.00 | 0.97 | 0.86 | 0.79 | 0.78 | 0.78 | 0.78 | 0.78 | 0.76 | 0.72 | 0.71 | 0.67 |
|  | 65 |  |  |  |  | 1.00 | 0.95 | 0.90 | 0.88 | 0.87 | 0.87 | 0.87 | 0.84 | 0.79 | 0.78 | 0.73 |
|  | 95 |  |  |  |  |  | 1.00 | 0.98 | 0.98 | 0.97 | 0.95 | 0.93 | 0.90 | 0.85 | 0.84 | 0.80 |
|  | 125 |  |  |  |  |  |  | 1.00 | 1.00 | 0.99 | 0.98 | 0.95 | 0.92 | 0.88 | 0.88 | 0.84 |
|  | 140 |  |  |  |  |  |  |  | 1.00 | 1.00 | 0.99 | 0.96 | 0.93 | 0.90 | 0.89 | 0.86 |

| **Traits** | **DIM** | **5** | **7** | **35** | **50** | **65** | **95** | **125** | **140** | **155** | **185** | **215** | **245** | **275** | **280** | **305** |
| --- | --- | --- | --- | --- | --- | --- | --- | --- | --- | --- | --- | --- | --- | --- | --- | --- |
| FP | 155 |  |  |  |  |  |  |  |  | 1.00 | 0.99 | 0.97 | 0.94 | 0.91 | 0.90 | 0.87 |
|  | 185 |  |  |  |  |  |  |  |  |  | 1.00 | 0.99 | 0.97 | 0.94 | 0.93 | 0.90 |
|  | 215 |  |  |  |  |  |  |  |  |  |  | 1.00 | 0.99 | 0.96 | 0.96 | 0.93 |
|  | 245 |  |  |  |  |  |  |  |  |  |  |  | 1.00 | 0.99 | 0.98 | 0.96 |
|  | 275 |  |  |  |  |  |  |  |  |  |  |  |  | 1.00 | 1.00 | 0.99 |
|  | 280 |  |  |  |  |  |  |  |  |  |  |  |  |  | 1.00 | 0.99 |
|  | 305 |  |  |  |  |  |  |  |  |  |  |  |  |  |  | 1.00 |
| FY | 5 | 1.00 | 1.00 | 0.68 | 0.43 | 0.27 | 0.16 | 0.15 | 0.14 | 0.12 | 0.08 | 0.04 | 0.05 | 0.11 | 0.13 | 0.16 |
|  | 7 |  | 1.00 | 0.71 | 0.47 | 0.31 | 0.20 | 0.18 | 0.17 | 0.15 | 0.10 | 0.06 | 0.07 | 0.13 | 0.14 | 0.17 |
|  | 35 |  |  | 1.00 | 0.95 | 0.87 | 0.75 | 0.65 | 0.61 | 0.58 | 0.52 | 0.47 | 0.42 | 0.36 | 0.35 | 0.29 |
|  | 50 |  |  |  | 1.00 | 0.98 | 0.89 | 0.79 | 0.75 | 0.72 | 0.66 | 0.61 | 0.54 | 0.43 | 0.41 | 0.32 |
|  | 65 |  |  |  |  | 1.00 | 0.96 | 0.88 | 0.84 | 0.80 | 0.74 | 0.69 | 0.60 | 0.48 | 0.46 | 0.36 |
|  | 95 |  |  |  |  |  | 1.00 | 0.97 | 0.95 | 0.92 | 0.86 | 0.79 | 0.71 | 0.59 | 0.56 | 0.47 |
|  | 125 |  |  |  |  |  |  | 1.00 | 1.00 | 0.98 | 0.94 | 0.87 | 0.79 | 0.68 | 0.66 | 0.57 |
|  | 140 |  |  |  |  |  |  |  | 1.00 | 1.00 | 0.96 | 0.90 | 0.83 | 0.73 | 0.71 | 0.62 |
|  | 155 |  |  |  |  |  |  |  |  | 1.00 | 0.98 | 0.94 | 0.87 | 0.77 | 0.76 | 0.67 |
|  | 185 |  |  |  |  |  |  |  |  |  | 1.00 | 0.98 | 0.94 | 0.85 | 0.83 | 0.74 |
|  | 215 |  |  |  |  |  |  |  |  |  |  | 1.00 | 0.98 | 0.91 | 0.89 | 0.81 |
|  | 245 |  |  |  |  |  |  |  |  |  |  |  | 1.00 | 0.97 | 0.96 | 0.90 |
|  | 275 |  |  |  |  |  |  |  |  |  |  |  |  | 1.00 | 1.00 | 0.98 |
|  | 280 |  |  |  |  |  |  |  |  |  |  |  |  |  | 1.00 | 0.99 |
|  | 305 |  |  |  |  |  |  |  |  |  |  |  |  |  |  | 1.00 |
|  | 5 | 1.00 | 1.00 | 0.64 | 0.42 | 0.33 | 0.35 | 0.41 | 0.42 | 0.41 | 0.37 | 0.34 | 0.34 | 0.37 | 0.38 | 0.36 |
| PP | 7 |  | 1.00 | 0.68 | 0.46 | 0.37 | 0.39 | 0.44 | 0.44 | 0.43 | 0.39 | 0.36 | 0.36 | 0.39 | 0.39 | 0.37 |
|  | 35 |  |  | 1.00 | 0.96 | 0.91 | 0.83 | 0.75 | 0.72 | 0.69 | 0.65 | 0.62 | 0.59 | 0.54 | 0.53 | 0.48 |
|  | 50 |  |  |  | 1.00 | 0.99 | 0.91 | 0.82 | 0.78 | 0.75 | 0.72 | 0.69 | 0.65 | 0.59 | 0.57 | 0.52 |
| **Traits** | **DIM** | **5** | **7** | **35** | **50** | **65** | **95** | **125** | **140** | **155** | **185** | **215** | **245** | **275** | **280** | **305** |
| PP | 65 |  |  |  |  | 1.00 | 0.96 | 0.87 | 0.84 | 0.81 | 0.78 | 0.76 | 0.72 | 0.65 | 0.64 | 0.58 |
|  | 95 |  |  |  |  |  | 1.00 | 0.97 | 0.95 | 0.94 | 0.91 | 0.89 | 0.85 | 0.79 | 0.78 | 0.73 |
|  | 125 |  |  |  |  |  |  | 1.00 | 1.00 | 0.99 | 0.97 | 0.95 | 0.92 | 0.87 | 0.86 | 0.81 |
|  | 140 |  |  |  |  |  |  |  | 1.00 | 1.00 | 0.99 | 0.97 | 0.94 | 0.89 | 0.88 | 0.83 |
|  | 155 |  |  |  |  |  |  |  |  | 1.00 | 0.99 | 0.98 | 0.95 | 0.90 | 0.89 | 0.84 |
|  | 185 |  |  |  |  |  |  |  |  |  | 1.00 | 0.99 | 0.97 | 0.93 | 0.92 | 0.87 |
|  | 215 |  |  |  |  |  |  |  |  |  |  | 1.00 | 0.99 | 0.95 | 0.94 | 0.90 |
|  | 245 |  |  |  |  |  |  |  |  |  |  |  | 1.00 | 0.98 | 0.98 | 0.95 |
|  | 275 |  |  |  |  |  |  |  |  |  |  |  |  | 1.00 | 1.00 | 0.99 |
|  | 280 |  |  |  |  |  |  |  |  |  |  |  |  |  | 1.00 | 0.99 |
|  | 305 |  |  |  |  |  |  |  |  |  |  |  |  |  |  | 1.00 |
| PY | 5 | 1.00 | 1.00 | 0.68 | 0.43 | 0.27 | 0.16 | 0.15 | 0.14 | 0.12 | 0.08 | 0.04 | 0.05 | 0.11 | 0.13 | 0.16 |
|  | 7 |  | 1.00 | 0.71 | 0.47 | 0.31 | 0.20 | 0.18 | 0.17 | 0.15 | 0.10 | 0.06 | 0.07 | 0.13 | 0.14 | 0.17 |
|  | 35 |  |  | 1.00 | 0.95 | 0.87 | 0.75 | 0.65 | 0.61 | 0.58 | 0.52 | 0.47 | 0.42 | 0.36 | 0.35 | 0.29 |
|  | 50 |  |  |  | 1.00 | 0.98 | 0.89 | 0.79 | 0.75 | 0.72 | 0.66 | 0.61 | 0.54 | 0.43 | 0.41 | 0.32 |
|  | 65 |  |  |  |  | 1.00 | 0.96 | 0.88 | 0.84 | 0.80 | 0.74 | 0.69 | 0.60 | 0.48 | 0.46 | 0.36 |
|  | 95 |  |  |  |  |  | 1.00 | 0.97 | 0.95 | 0.92 | 0.86 | 0.79 | 0.71 | 0.59 | 0.56 | 0.47 |
|  | 125 |  |  |  |  |  |  | 1.00 | 1.00 | 0.98 | 0.94 | 0.87 | 0.79 | 0.68 | 0.66 | 0.57 |
|  | 140 |  |  |  |  |  |  |  | 1.00 | 1.00 | 0.96 | 0.90 | 0.83 | 0.73 | 0.71 | 0.62 |
|  | 155 |  |  |  |  |  |  |  |  | 1.00 | 0.98 | 0.94 | 0.87 | 0.77 | 0.76 | 0.67 |
|  | 185 |  |  |  |  |  |  |  |  |  | 1.00 | 0.98 | 0.94 | 0.85 | 0.83 | 0.74 |
|  | 215 |  |  |  |  |  |  |  |  |  |  | 1.00 | 0.98 | 0.91 | 0.89 | 0.81 |
|  | 245 |  |  |  |  |  |  |  |  |  |  |  | 1.00 | 0.97 | 0.96 | 0.90 |
|  | 275 |  |  |  |  |  |  |  |  |  |  |  |  | 1.00 | 1.00 | 0.98 |
|  | 280 |  |  |  |  |  |  |  |  |  |  |  |  |  | 1.00 | 0.99 |
|  | 305 |  |  |  |  |  |  |  |  |  |  |  |  |  |  | 1.00 |

| **Traits** | **DIM** | **5** | **7** | **35** | **50** | **65** | **95** | **125** | **140** | **155** | **185** | **215** | **245** | **275** | **280** | **305** |
| --- | --- | --- | --- | --- | --- | --- | --- | --- | --- | --- | --- | --- | --- | --- | --- | --- |
| SCS | 5 | 1.00 | 1.00 | 0.69 | 0.38 | 0.18 | 0.05 | 0.05 | 0.05 | 0.04 | 0.01 | -0.02 | -0.02 | 0.02 | 0.02 | 0.04 |
|  | 7 |  | 1.00 | 0.72 | 0.42 | 0.22 | 0.09 | 0.08 | 0.08 | 0.07 | 0.03 | 0.00 | 0.01 | 0.04 | 0.05 | 0.06 |
|  | 35 |  |  | 1.00 | 0.93 | 0.83 | 0.72 | 0.67 | 0.65 | 0.63 | 0.59 | 0.56 | 0.54 | 0.54 | 0.54 | 0.51 |
|  | 50 |  |  |  | 1.00 | 0.97 | 0.91 | 0.86 | 0.84 | 0.82 | 0.78 | 0.75 | 0.73 | 0.71 | 0.71 | 0.68 |
|  | 65 |  |  |  |  | 1.00 | 0.98 | 0.94 | 0.92 | 0.90 | 0.87 | 0.84 | 0.82 | 0.79 | 0.78 | 0.75 |
|  | 95 |  |  |  |  |  | 1.00 | 0.99 | 0.98 | 0.96 | 0.94 | 0.91 | 0.89 | 0.86 | 0.86 | 0.82 |
|  | 125 |  |  |  |  |  |  | 1.00 | 1.00 | 0.99 | 0.97 | 0.95 | 0.93 | 0.91 | 0.91 | 0.88 |
|  | 140 |  |  |  |  |  |  |  | 1.00 | 1.00 | 0.99 | 0.97 | 0.95 | 0.93 | 0.92 | 0.89 |
|  | 155 |  |  |  |  |  |  |  |  | 1.00 | 0.99 | 0.98 | 0.96 | 0.94 | 0.94 | 0.91 |
|  | 185 |  |  |  |  |  |  |  |  |  | 1.00 | 1.00 | 0.99 | 0.97 | 0.96 | 0.93 |
|  | 215 |  |  |  |  |  |  |  |  |  |  | 1.00 | 1.00 | 0.98 | 0.98 | 0.95 |
|  | 245 |  |  |  |  |  |  |  |  |  |  |  | 1.00 | 0.99 | 0.99 | 0.97 |
|  | 275 |  |  |  |  |  |  |  |  |  |  |  |  | 1.00 | 1.00 | 0.99 |
|  | 280 |  |  |  |  |  |  |  |  |  |  |  |  |  | 1.00 | 0.99 |
|  | 305 |  |  |  |  |  |  |  |  |  |  |  |  |  |  | 1.00 |

**Table S2.** The number of significant SNPs for different days in milk of milk production traits detected by GWAS based on FarmCPU

| **Traits** | **5** | **7** | **35** | **50** | **65** | **95** | **125** | **140** | **155** | **185** | **215** | **245** | **275** | **280** | **305** | **Total** |
| --- | --- | --- | --- | --- | --- | --- | --- | --- | --- | --- | --- | --- | --- | --- | --- | --- |
| MY | 146 | 139 | 137 | 114 | 87 | 89 | 95 | 85 | 105 | 77 | 70 | 84 | 74 | 73 | 81 | 984 |
| FP | 190 | 188 | 113 | 105 | 109 | 98 | 102 | 114 | 111 | 115 | 100 | 115 | 109 | 106 | 109 | 1150 |
| FY | 158 | 135 | 130 | 141 | 161 | 113 | 118 | 126 | 125 | 99 | 110 | 99 | 92 | 90 | 87 | 1291 |
| PP | 150 | 131 | 97 | 109 | 127 | 139 | 143 | 114 | 133 | 139 | 130 | 118 | 128 | 135 | 125 | 1229 |
| PY | 178 | 181 | 140 | 116 | 93 | 68 | 66 | 66 | 80 | 76 | 77 | 79 | 67 | 60 | 76 | 1018 |
| SCS | 297 | 275 | 201 | 170 | 146 | 114 | 111 | 119 | 103 | 105 | 112 | 110 | 100 | 89 | 119 | 1477 |

**Table S3.** The number of significant SNPs for different days in milk of milk production traits detected by GWAS based on MLM

| **Traits** | **5** | **7** | **35** | **50** | **65** | **95** | **125** | **140** | **155** | **185** | **215** | **245** | **275** | **280** | **305** | **Total** |
| --- | --- | --- | --- | --- | --- | --- | --- | --- | --- | --- | --- | --- | --- | --- | --- | --- |
| MY | 44 | 37 | 1 | 10 | 59 | 91 | 89 | 89 | 86 | 87 | 81 | 153 | 170 | 169 | 147 | 279 |
| FP | 1 | 0 | 216 | 323 | 326 | 331 | 365 | 378 | 386 | 400 | 424 | 425 | 419 | 416 | 414 | 429 |
| FY | 6 | 6 | 8 | 0 | 3 | 3 | 13 | 14 | 15 | 15 | 15 | 13 | 4 | 2 | 2 | 36 |
| PP | 1 | 1 | 0 | 5 | 42 | 144 | 149 | 154 | 150 | 151 | 163 | 153 | 150 | 149 | 146 | 175 |
| PY | 36 | 31 | 2 | 1 | 3 | 24 | 32 | 36 | 41 | 40 | 22 | 8 | 2 | 0 | 0 | 85 |
| SCS | 13 | 13 | 2 | 1 | 2 | 9 | 9 | 10 | 11 | 15 | 18 | 14 | 6 | 5 | 5 | 42 |

**Table S4.** The number of common SNPs for different days in milk of milk production traits detected by GWAS based on FarmCPU and MLM

| **Traits** | **5** | **7** | **35** | **50** | **65** | **95** | **125** | **140** | **155** | **185** | **215** | **245** | **275** | **280** | **305** | **Total** |
| --- | --- | --- | --- | --- | --- | --- | --- | --- | --- | --- | --- | --- | --- | --- | --- | --- |
| MY | 6 | 6 | 1 | 2 | 3 | 3 | 5 | 4 | 2 | 4 | 2 | 2 | 1 | 2 | 1 | 44 |
| FP | 1 | 0 | 1 | 1 | 1 | 1 | 3 | 3 | 3 | 3 | 4 | 3 | 2 | 2 | 2 | 30 |
| FY | 1 | 1 | 1 | 0 | 2 | 1 | 2 | 2 | 3 | 1 | 1 | 2 | 0 | 1 | 2 | 20 |
| PP | 0 | 0 | 0 | 0 | 0 | 1 | 1 | 1 | 1 | 1 | 1 | 1 | 3 | 3 | 3 | 16 |
| PY | 6 | 5 | 1 | 0 | 1 | 1 | 1 | 2 | 2 | 3 | 2 | 1 | 1 | 0 | 0 | 26 |
| SCS | 5 | 4 | 2 | 1 | 2 | 2 | 4 | 3 | 2 | 3 | 3 | 3 | 2 | 2 | 3 | 41 |

**Table S5.** The total number of significant SNPs for different days in milk of milk production traits detected by GWAS based on FarmCPU and MLM

| **Traits** | **5** | **7** | **35** | **50** | **65** | **95** | **125** | **140** | **155** | **185** | **215** | **245** | **275** | **280** | **305** | **Total** |
| --- | --- | --- | --- | --- | --- | --- | --- | --- | --- | --- | --- | --- | --- | --- | --- | --- |
| MY | 184 | 170 | 137 | 122 | 143 | 177 | 179 | 170 | 189 | 160 | 149 | 235 | 243 | 240 | 227 | 1241 |
| FP | 190 | 188 | 328 | 427 | 434 | 428 | 464 | 489 | 494 | 512 | 520 | 537 | 526 | 520 | 521 | 1568 |
| FY | 163 | 140 | 137 | 141 | 162 | 115 | 129 | 138 | 137 | 113 | 124 | 110 | 96 | 91 | 87 | 1316 |
| PP | 151 | 132 | 97 | 114 | 169 | 282 | 291 | 267 | 282 | 289 | 292 | 270 | 275 | 281 | 268 | 1399 |
| PY | 208 | 207 | 141 | 117 | 95 | 91 | 97 | 100 | 119 | 113 | 97 | 86 | 68 | 60 | 76 | 1087 |
| SCS | 305 | 284 | 201 | 170 | 146 | 121 | 116 | 126 | 112 | 117 | 127 | 121 | 104 | 92 | 121 | 1503 |

**Table S6.** Enrichment analysis for the genes associated with the FP of TD7, 35, 50, 140 and 280

| **DIM** | **GO accession number** | **GO terms** | **No. Genes** | | **p-value** |
| --- | --- | --- | --- | --- | --- |
| 7 | GO:0023057 | negative regulation of signaling | | 35 | 2.52E-03 |
|  | GO:0023051 | regulation of signaling | | 71 | 5.47E-03 |
|  | GO:0032947 | protein complex scaffold | | 4 | 7.44E-03 |
|  | GO:0009719 | response to endogenous stimulus | | 34 | 8.06E-03 |
|  | GO:0019898 | extrinsic component of membrane | | 12 | 1.38E-02 |
|  | GO:0044406 | adhesion of symbiont to host | | 3 | 1.51E-02 |
|  | GO:0048585 | negative regulation of response to stimulus | | 36 | 1.78E-02 |
|  | GO:0048583 | regulation of response to stimulus | | 78 | 1.95E-02 |
|  | GO:0098552 | side of membrane | | 15 | 2.06E-02 |
|  | GO:0044763 | single-organism cellular process | | 250 | 2.50E-02 |
|  | GO:0048519 | negative regulation of biological process | | 96 | 2.50E-02 |
|  | GO:0016247 | channel regulator activity | | 7 | 2.77E-02 |
|  | GO:0043167 | ion binding | | 91 | 3.17E-02 |
|  | GO:0065008 | regulation of biological quality | | 74 | 4.19E-02 |
|  | GO:0044700 | single organism signaling | | 129 | 4.21E-02 |
| 35 | GO:0006950 | response to stress | | 53 | 1.60E-03 |
|  | GO:0002252 | immune effector process | | 17 | 4.18E-03 |
|  | GO:0009628 | response to abiotic stimulus | | 18 | 7.57E-03 |
|  | GO:0003823 | antigen binding | | 4 | 9.73E-03 |
|  | GO:0042267 | natural killer cell mediated cytotoxicity | | 4 | 1.07E-02 |
|  | GO:0019882 | antigen processing and presentation | | 6 | 1.10E-02 |
|  | GO:0006955 | immune response | | 22 | 1.73E-02 |
|  | GO:0051183 | vitamin transporter activity | | 3 | 2.03E-02 |
|  | GO:0043167 | ion binding | | 56 | 2.65E-02 |
|  | GO:0019898 | extrinsic component of membrane | | 8 | 2.86E-02 |
|  | GO:0022402 | cell cycle process | | 20 | 3.09E-02 |
|  | GO:0098552 | side of membrane | | 10 | 3.21E-02 |
|  | GO:0000785 | chromatin | | 12 | 3.62E-02 |
|  | GO:0009975 | cyclase activity | | 3 | 3.70E-02 |
|  | GO:0044455 | mitochondrial membrane part | | 7 | 4.00E-02 |
|  | GO:0001909 | leukocyte mediated cytotoxicity | | 4 | 4.45E-02 |
|  | GO:0009719 | response to endogenous stimulus | | 20 | 4.61E-02 |
|  | GO:0012505 | endomembrane system | | 47 | 4.82E-02 |
| 50 | GO:0044707 | single-multicellular organism process | | 86 | 1.71E-02 |
|  | GO:0050793 | regulation of developmental process | | 37 | 2.54E-02 |
|  | GO:0009628 | response to abiotic stimulus | | 18 | 2.69E-02 |
|  | GO:0003700 | transcription factor activity, sequence-specific DNA binding | | 23 | 2.93E-02 |
|  | GO:0019953 | sexual reproduction | | 15 | 3.30E-02 |
|  | GO:0044703 | multi-organism reproductive process | | 16 | 3.98E-02 |
|  | GO:0022414 | reproductive process | | 22 | 4.99E-02 |
| 140 | GO:0000785 | chromatin | | 15 | 6.41E-03 |
|  | GO:0023051 | regulation of signaling | | 48 | 7.76E-03 |
|  | GO:0050793 | regulation of developmental process | | 37 | 1.01E-02 |
|  | GO:0051094 | positive regulation of developmental process | | 22 | 1.39E-02 |
|  | GO:0032993 | protein-DNA complex | | 8 | 1.77E-02 |
|  | GO:0051240 | positive regulation of multicellular organismal process | | 26 | 1.88E-02 |
|  | GO:0043234 | protein complex | | 67 | 2.87E-02 |
|  | GO:0051239 | regulation of multicellular organismal process | | 41 | 3.35E-02 |
|  | GO:0048583 | regulation of response to stimulus | | 51 | 3.78E-02 |
|  | GO:0044297 | cell body | | 8 | 3.82E-02 |
|  | GO:0019953 | sexual reproduction | | 14 | 4.20E-02 |
|  | GO:0042221 | response to chemical | | 50 | 4.32E-02 |
|  | GO:0044424 | intracellular part | | 178 | 4.88E-02 |
| 280 | GO:0044456 | synapse part | | 12 | 2.15E-02 |
|  | GO:0098794 | postsynapse | | 8 | 2.63E-02 |
|  | GO:0016740 | transferase activity | | 43 | 4.15E-02 |

**Table S7.** Enrichment analysis for the genes associated with the FY of D7, 35, 50, 140 and 280

| **DIM** | **GO accession number** | **GO terms** | **No. Genes** | | **p-value** |
| --- | --- | --- | --- | --- | --- |
| 7 | GO:0042995 | cell projection | | 24 | 9.96E-03 |
|  | GO:0048583 | regulation of response to stimulus | | 43 | 1.15E-02 |
|  | GO:0097458 | neuron part | | 18 | 1.27E-02 |
|  | GO:0033036 | macromolecule localization | | 34 | 1.80E-02 |
|  | GO:0009975 | cyclase activity | | 3 | 2.77E-02 |
|  | GO:0002682 | regulation of immune system process | | 18 | 2.95E-02 |
|  | GO:0044297 | cell body | | 7 | 3.40E-02 |
|  | GO:0048584 | positive regulation of response to stimulus | | 24 | 4.08E-02 |
|  | GO:0051234 | establishment of localization | | 49 | 4.32E-02 |
| 35 | GO:0099513 | polymeric cytoskeletal fiber | | 19 | 1.21E-04 |
|  | GO:0003008 | system process | | 32 | 1.16E-02 |
|  | GO:0044767 | single-organism developmental process | | 73 | 1.61E-02 |
|  | GO:0050795 | regulation of behavior | | 4 | 1.68E-02 |
|  | GO:0048646 | anatomical structure formation involved in morphogenesis | | 22 | 2.57E-02 |
|  | GO:0044459 | plasma membrane part | | 37 | 3.47E-02 |
|  | GO:0009653 | anatomical structure morphogenesis | | 40 | 3.83E-02 |
|  | GO:0048856 | anatomical structure development | | 70 | 4.46E-02 |
| 50 | GO:0012505 | endomembrane system | | 56 | 8.19E-04 |
|  | GO:0031090 | organelle membrane | | 38 | 2.58E-03 |
|  | GO:0022857 | transmembrane transporter activity | | 24 | 3.22E-03 |
|  | GO:0022892 | substrate-specific transporter activity | | 25 | 3.82E-03 |
|  | GO:0007631 | feeding behavior | | 5 | 1.16E-02 |
|  | GO:0097458 | neuron part | | 20 | 1.51E-02 |
|  | GO:1902578 | single-organism localization | | 43 | 1.53E-02 |
|  | GO:0044710 | single-organism metabolic process | | 49 | 1.70E-02 |
|  | GO:0009719 | response to endogenous stimulus | | 20 | 1.77E-02 |
|  | GO:0048583 | regulation of response to stimulus | | 45 | 2.03E-02 |
|  | GO:0023056 | positive regulation of signaling | | 23 | 2.08E-02 |
|  | GO:0044425 | membrane part | | 101 | 2.43E-02 |
|  | GO:0044459 | plasma membrane part | | 36 | 2.62E-02 |
|  | GO:0005085 | guanyl-nucleotide exchange factor activity | | 7 | 3.63E-02 |
|  | GO:0031224 | intrinsic component of membrane | | 88 | 3.78E-02 |
|  | GO:0030234 | enzyme regulator activity | | 17 | 4.02E-02 |
|  | GO:0044763 | single-organism cellular process | | 135 | 4.20E-02 |
| 140 | GO:0097458 | neuron part | | 28 | 2.94E-04 |
|  | GO:0033036 | macromolecule localization | | 51 | 2.00E-03 |
|  | GO:0042995 | cell projection | | 31 | 9.90E-03 |
|  | GO:0044297 | cell body | | 9 | 2.02E-02 |
|  | GO:0051641 | cellular localization | | 42 | 3.15E-02 |
|  | GO:1902578 | single-organism localization | | 53 | 3.18E-02 |
|  | GO:0051234 | establishment of localization | | 69 | 3.60E-02 |
| 280 | GO:0048870 | cell motility | | 19 | 8.61E-04 |
|  | GO:0051674 | localization of cell | | 19 | 8.61E-04 |
|  | GO:0042330 | taxis | | 11 | 1.69E-03 |
|  | GO:0040012 | regulation of locomotion | | 13 | 2.27E-03 |
|  | GO:0032879 | regulation of localization | | 25 | 6.98E-03 |
|  | GO:0009605 | response to external stimulus | | 21 | 1.63E-02 |
|  | GO:0048583 | regulation of response to stimulus | | 31 | 1.97E-02 |
|  | GO:0009653 | anatomical structure morphogenesis | | 25 | 3.48E-02 |
|  | GO:0048519 | negative regulation of biological process | | 36 | 4.31E-02 |
|  | GO:0044459 | plasma membrane part | | 23 | 4.36E-02 |
|  | GO:0040017 | positive regulation of locomotion | | 7 | 4.64E-02 |

**Table S8.** Enrichment analysis for the genes associated with MY of TD7, 35, 50, 140 and 280

| **DIM** | **GO accession number** | | **GO terms** | **No. Genes** | | **p-value** |
| --- | --- | --- | --- | --- | --- | --- |
| 7 | GO:0005622 | intracellular | | | 198 | 6.18E-03 |
|  | GO:0044424 | intracellular part | | | 191 | 6.20E-03 |
|  | GO:0097458 | neuron part | | | 22 | 1.62E-02 |
|  | GO:0043229 | intracellular organelle | | | 166 | 1.71E-02 |
|  | GO:0045927 | positive regulation of growth | | | 8 | 2.05E-02 |
|  | GO:0044464 | cell part | | | 227 | 2.18E-02 |
|  | GO:0016740 | transferase activity | | | 43 | 4.44E-02 |
| 35 | GO:0043227 | membrane-bounded organelle | | | 187 | 1.02E-02 |
|  | GO:0097159 | organic cyclic compound binding | | | 100 | 2.35E-02 |
|  | GO:0048037 | cofactor binding | | | 10 | 2.40E-02 |
|  | GO:0043167 | ion binding | | | 68 | 2.56E-02 |
|  | GO:0044710 | single-organism metabolic process | | | 65 | 2.67E-02 |
|  | GO:0098644 | complex of collagen trimers | | | 3 | 2.94E-02 |
|  | GO:1901363 | heterocyclic compound binding | | | 97 | 4.31E-02 |
| 50 | GO:0042995 | cell projection | | | 30 | 3.21E-04 |
|  | GO:0097458 | neuron part | | | 23 | 4.47E-04 |
|  | GO:0023057 | negative regulation of signaling | | | 22 | 3.16E-03 |
|  | GO:0048585 | negative regulation of response to stimulus | | | 24 | 5.19E-03 |
|  | GO:0044463 | cell projection part | | | 15 | 8.82E-03 |
|  | GO:0098590 | plasma membrane region | | | 15 | 9.68E-03 |
|  | GO:0023051 | regulation of signaling | | | 40 | 1.77E-02 |
|  | GO:0044877 | macromolecular complex binding | | | 18 | 1.89E-02 |
|  | GO:0045177 | apical part of cell | | | 8 | 2.25E-02 |
|  | GO:0044459 | plasma membrane part | | | 32 | 4.59E-02 |
|  | GO:0003700 | transcription factor activity, sequence-specific DNA binding | | | 19 | 4.95E-02 |
| 140 | GO:0097458 | neuron part | | | 22 | 2.04E-03 |
|  | GO:0044459 | plasma membrane part | | | 39 | 2.57E-03 |
|  | GO:0038023 | signaling receptor activity | | | 33 | 5.69E-03 |
|  | GO:0004872 | receptor activity | | | 35 | 9.36E-03 |
|  | GO:0044700 | single organism signaling | | | 78 | 4.09E-02 |
| 280 | GO:0097458 | neuron part | | | 16 | 5.59E-03 |
|  | GO:0042995 | cell projection | | | 20 | 8.80E-03 |
|  | GO:0044459 | plasma membrane part | | | 27 | 1.10E-02 |
|  | GO:0044424 | intracellular part | | | 108 | 4.52E-02 |

**Table S9.** Enrichment analysis for the genes associated with PP of TD7, 35, 50, 140 and 280

| **DIM** | **GO accession number** | | **GO terms** | **No. Genes** | | **p-value** |
| --- | --- | --- | --- | --- | --- | --- |
| 7 | GO:0003008 | system process | | | 47 | 3.28E-06 |
|  | GO:0038023 | signaling receptor activity | | | 46 | 7.42E-05 |
|  | GO:0004872 | receptor activity | | | 49 | 1.29E-04 |
|  | GO:0051606 | detection of stimulus | | | 17 | 1.41E-04 |
|  | GO:0099513 | polymeric cytoskeletal fiber | | | 17 | 2.27E-03 |
|  | GO:0044700 | single organism signaling | | | 96 | 5.86E-03 |
|  | GO:0050794 | regulation of cellular process | | | 152 | 6.20E-03 |
|  | GO:0071944 | cell periphery | | | 81 | 1.12E-02 |
|  | GO:0005886 | plasma membrane | | | 79 | 1.25E-02 |
|  | GO:0043228 | non-membrane-bounded organelle | | | 65 | 1.86E-02 |
|  | GO:0051716 | cellular response to stimulus | | | 103 | 2.89E-02 |
| 35 | GO:0048589 | developmental growth | | | 14 | 1.98E-03 |
|  | GO:0045927 | positive regulation of growth | | | 8 | 3.16E-03 |
|  | GO:0051241 | negative regulation of multicellular organismal process | | | 17 | 9.51E-03 |
|  | GO:0040008 | regulation of growth | | | 12 | 1.13E-02 |
|  | GO:0051674 | localization of cell | | | 20 | 1.40E-02 |
|  | GO:0048870 | cell motility | | | 20 | 1.40E-02 |
|  | GO:0044707 | single-multicellular organism process | | | 62 | 1.60E-02 |
|  | GO:0048585 | negative regulation of response to stimulus | | | 20 | 1.79E-02 |
|  | GO:0044767 | single-organism developmental process | | | 58 | 2.04E-02 |
|  | GO:0040012 | regulation of locomotion | | | 13 | 2.79E-02 |
|  | GO:0043167 | ion binding | | | 49 | 2.84E-02 |
|  | GO:0048856 | anatomical structure development | | | 57 | 2.96E-02 |
|  | GO:0051919 | positive regulation of fibrinolysis | | | 2 | 3.38E-02 |
|  | GO:0006807 | nitrogen compound metabolic process | | | 67 | 4.03E-02 |
|  | GO:0008283 | cell proliferation | | | 22 | 4.05E-02 |
|  | GO:0051239 | regulation of multicellular organismal process | | | 31 | 4.61E-02 |
|  | GO:0051093 | negative regulation of developmental process | | | 12 | 4.75E-02 |
| 50 | GO:0005622 | intracellular | | | 202 | 2.85E-03 |
|  | GO:0048585 | negative regulation of response to stimulus | | | 27 | 7.68E-03 |
|  | GO:0048519 | negative regulation of biological process | | | 67 | 1.32E-02 |
|  | GO:0048523 | negative regulation of cellular process | | | 63 | 1.52E-02 |
|  | GO:0006807 | nitrogen compound metabolic process | | | 93 | 1.63E-02 |
|  | GO:0044424 | intracellular part | | | 190 | 2.09E-02 |
|  | GO:0003700 | transcription factor activity, sequence-specific DNA binding | | | 24 | 2.34E-02 |
|  | GO:0044449 | contractile fiber part | | | 7 | 2.46E-02 |
|  | GO:0023057 | negative regulation of signaling | | | 22 | 2.52E-02 |
|  | GO:0044767 | single-organism developmental process | | | 75 | 4.53E-02 |
| 140 | GO:1901363 | heterocyclic compound binding | | | 94 | 3.13E-03 |
|  | GO:0044237 | cellular metabolic process | | | 130 | 3.24E-03 |
|  | GO:0097159 | organic cyclic compound binding | | | 94 | 4.66E-03 |
|  | GO:0006807 | nitrogen compound metabolic process | | | 90 | 5.57E-03 |
|  | GO:0030234 | enzyme regulator activity | | | 21 | 6.88E-03 |
|  | GO:0044238 | primary metabolic process | | | 125 | 1.50E-02 |
|  | GO:0016874 | ligase activity | | | 9 | 2.13E-02 |
|  | GO:0048523 | negative regulation of cellular process | | | 58 | 2.47E-02 |
|  | GO:0097367 | carbohydrate derivative binding | | | 39 | 3.11E-02 |
|  | GO:0071704 | organic substance metabolic process | | | 129 | 3.15E-02 |
|  | GO:0043227 | membrane-bounded organelle | | | 152 | 3.30E-02 |
| 280 | GO:0009892 | negative regulation of metabolic process | | | 54 | 6.45E-06 |
|  | GO:0099513 | polymeric cytoskeletal fiber | | | 23 | 4.32E-05 |
|  | GO:0048523 | negative regulation of cellular process | | | 80 | 6.94E-05 |
|  | GO:0048519 | negative regulation of biological process | | | 84 | 7.83E-05 |
|  | GO:0005615 | extracellular space | | | 36 | 7.21E-04 |
|  | GO:0044707 | single-multicellular organism process | | | 94 | 4.52E-03 |
|  | GO:0040012 | regulation of locomotion | | | 20 | 4.92E-03 |
|  | GO:0051239 | regulation of multicellular organismal process | | | 49 | 6.89E-03 |
|  | GO:0008283 | cell proliferation | | | 34 | 9.06E-03 |
|  | GO:0051241 | negative regulation of multicellular organismal process | | | 23 | 9.68E-03 |
|  | GO:0048583 | regulation of response to stimulus | | | 60 | 1.02E-02 |
|  | GO:0023056 | positive regulation of signaling | | | 30 | 1.27E-02 |
|  | GO:0065009 | regulation of molecular function | | | 43 | 1.28E-02 |
|  | GO:0050793 | regulation of developmental process | | | 40 | 1.43E-02 |
|  | GO:0044421 | extracellular region part | | | 74 | 1.47E-02 |
|  | GO:0009605 | response to external stimulus | | | 38 | 1.54E-02 |
|  | GO:0023051 | regulation of signaling | | | 51 | 1.81E-02 |
|  | GO:0051674 | localization of cell | | | 27 | 1.90E-02 |
|  | GO:0048870 | cell motility | | | 27 | 1.90E-02 |
|  | GO:0007269 | neurotransmitter secretion | | | 6 | 1.91E-02 |
|  | GO:0040013 | negative regulation of locomotion | | | 9 | 1.93E-02 |
|  | GO:0016043 | cellular component organization | | | 96 | 2.03E-02 |
|  | GO:0048584 | positive regulation of response to stimulus | | | 34 | 2.51E-02 |
|  | GO:0007626 | locomotory behavior | | | 8 | 2.61E-02 |
|  | GO:0005578 | proteinaceous extracellular matrix | | | 11 | 2.86E-02 |
|  | GO:0009653 | anatomical structure morphogenesis | | | 47 | 3.15E-02 |
|  | GO:0044703 | multi-organism reproductive process | | | 17 | 3.22E-02 |
|  | GO:0044767 | single-organism developmental process | | | 83 | 3.49E-02 |
|  | GO:0097458 | neuron part | | | 23 | 4.32E-02 |
|  | GO:0040008 | regulation of growth | | | 14 | 4.33E-02 |
|  | GO:0019953 | sexual reproduction | | | 15 | 4.89E-02 |

**Table S10.** Enrichment analysis for the genes associated with the PY of TD7, 35, 50, 140 and 280

| **DIM** | **GO accession number** | | **GO terms** | **No. Genes** | | **p-value** |
| --- | --- | --- | --- | --- | --- | --- |
| 7 | GO:0098796 | membrane protein complex | | | 34 | 7.95E-03 |
|  | GO:0048589 | developmental growth | | | 21 | 9.04E-03 |
|  | GO:0051606 | detection of stimulus | | | 17 | 9.45E-03 |
|  | GO:0044297 | cell body | | | 11 | 2.39E-02 |
|  | GO:0045927 | positive regulation of growth | | | 10 | 2.45E-02 |
|  | GO:0009605 | response to external stimulus | | | 48 | 4.44E-02 |
| 35 | GO:0044455 | mitochondrial membrane part | | | 8 | 1.07E-02 |
|  | GO:0043167 | ion binding | | | 56 | 1.35E-02 |
|  | GO:0005929 | cilium | | | 11 | 2.71E-02 |
|  | GO:0050900 | leukocyte migration | | | 8 | 2.73E-02 |
|  | GO:0042995 | cell projection | | | 24 | 3.72E-02 |
| 50 | GO:0042995 | cell projection | | | 27 | 1.92E-02 |
|  | GO:0051606 | detection of stimulus | | | 11 | 2.93E-02 |
|  | GO:0070161 | anchoring junction | | | 13 | 3.18E-02 |
|  | GO:0043167 | ion binding | | | 57 | 3.70E-02 |
|  | GO:0097458 | neuron part | | | 19 | 4.21E-02 |
| 140 | GO:0044237 | cellular metabolic process | | | 73 | 2.84E-02 |
|  | GO:0009058 | biosynthetic process | | | 46 | 3.57E-02 |
|  | GO:0044238 | primary metabolic process | | | 71 | 4.97E-02 |
| 280 | GO:0005549 | odorant binding | | | 11 | 4.24E-07 |
|  | GO:0099572 | postsynaptic specialization | | | 5 | 1.12E-03 |
|  | GO:0060076 | excitatory synapse | | | 5 | 1.83E-03 |
|  | GO:0098794 | postsynapse | | | 6 | 8.35E-03 |
|  | GO:0098590 | plasma membrane region | | | 10 | 1.44E-02 |
|  | GO:0044456 | synapse part | | | 7 | 3.08E-02 |

**Table S11.** Enrichment analysis for the genes associated with SCS of TD7, 35, 50, 140 and 280

| **DIM** | **GO accession number** | **GO terms** | **No. Genes** | | **p-value** |
| --- | --- | --- | --- | --- | --- |
| 7 | GO:0016787 | hydrolase activity | | 81 | 5.48E-03 |
|  | GO:0040012 | regulation of locomotion | | 27 | 1.23E-02 |
|  | GO:0000989 | transcription factor activity, transcription factor binding | | 20 | 1.56E-02 |
|  | GO:0044419 | interspecies interaction between organisms | | 23 | 2.33E-02 |
|  | GO:0044764 | multi-organism cellular process | | 22 | 2.38E-02 |
|  | GO:0030155 | regulation of cell adhesion | | 22 | 2.40E-02 |
|  | GO:0030234 | enzyme regulator activity | | 31 | 2.41E-02 |
| 35 | GO:0044708 | single-organism behavior | | 13 | 1.69E-02 |
|  | GO:0031252 | cell leading edge | | 11 | 2.69E-02 |
|  | GO:0008289 | lipid binding | | 17 | 3.88E-02 |
|  | GO:0030055 | cell-substrate junction | | 13 | 4.24E-02 |
|  | GO:0070161 | anchoring junction | | 16 | 4.73E-02 |
| 50 | GO:0051606 | detection of stimulus | | 22 | 3.58E-05 |
|  | GO:0003008 | system process | | 55 | 1.02E-04 |
|  | GO:0004872 | receptor activity | | 60 | 1.60E-04 |
|  | GO:0038023 | signaling receptor activity | | 54 | 3.35E-04 |
|  | GO:0034358 | plasma lipoprotein particle | | 5 | 2.64E-03 |
|  | GO:0042221 | response to chemical | | 79 | 2.68E-03 |
|  | GO:0042330 | taxis | | 20 | 2.70E-03 |
|  | GO:0032994 | protein-lipid complex | | 5 | 3.49E-03 |
|  | GO:0051716 | cellular response to stimulus | | 144 | 1.19E-02 |
|  | GO:0009605 | response to external stimulus | | 47 | 2.35E-02 |
|  | GO:0005886 | plasma membrane | | 103 | 3.77E-02 |
|  | GO:0023056 | positive regulation of signaling | | 35 | 4.00E-02 |
|  | GO:0044700 | single organism signaling | | 123 | 4.52E-02 |
| 140 | GO:0051606 | detection of stimulus | | 38 | 9.65E-19 |
|  | GO:0038023 | signaling receptor activity | | 67 | 3.62E-11 |
|  | GO:0004872 | receptor activity | | 70 | 2.26E-10 |
|  | GO:0003008 | system process | | 59 | 1.91E-09 |
|  | GO:0005886 | plasma membrane | | 100 | 2.12E-05 |
|  | GO:0071944 | cell periphery | | 101 | 3.57E-05 |
|  | GO:0044425 | membrane part | | 137 | 2.20E-04 |
|  | GO:0044700 | single organism signaling | | 113 | 3.13E-04 |
|  | GO:0042221 | response to chemical | | 69 | 4.05E-04 |
|  | GO:0031224 | intrinsic component of membrane | | 119 | 9.37E-04 |
|  | GO:0051716 | cellular response to stimulus | | 120 | 4.09E-03 |
|  | GO:0044763 | single-organism cellular process | | 193 | 1.73E-02 |
|  | GO:0050794 | regulation of cellular process | | 164 | 2.96E-02 |
|  | GO:0007626 | locomotory behavior | | 8 | 3.40E-02 |
|  | GO:0032947 | protein complex scaffold | | 3 | 3.94E-02 |
| 280 | GO:0016049 | cell growth | | 8 | 3.02E-02 |
|  | GO:0045927 | positive regulation of growth | | 6 | 3.16E-02 |
|  | GO:0048037 | cofactor binding | | 7 | 3.47E-02 |

**Table S12.** Pathways analysis for the genes associated with FP of TD7, 35, 50, 140 and 280

| **DIM** | **accession number** | **KEGG pathway** | **No. Genes** | | **p-value** |
| --- | --- | --- | --- | --- | --- |
| 7 | bta04010 | MAPK signaling pathway | | 17 | 2.04E-04 |
|  | bta05200 | Pathways in cancer | | 19 | 3.80E-03 |
|  | bta05030 | Cocaine addiction | | 6 | 4.79E-03 |
|  | bta04015 | Rap1 signaling pathway | | 12 | 9.18E-03 |
|  | bta04670 | Leukocyte transendothelial migration | | 8 | 1.60E-02 |
|  | bta04014 | Ras signaling pathway | | 12 | 1.75E-02 |
|  | bta05166 | HTLV-I infection | | 13 | 1.79E-02 |
|  | bta04360 | Axon guidance | | 8 | 2.31E-02 |
|  | bta05218 | Melanoma | | 6 | 2.32E-02 |
|  | bta04062 | Chemokine signaling pathway | | 10 | 2.44E-02 |
|  | bta04916 | Melanogenesis | | 7 | 2.45E-02 |
|  | bta04917 | Prolactin signaling pathway | | 6 | 2.58E-02 |
|  | bta04261 | Adrenergic signaling in cardiomyocytes | | 8 | 3.45E-02 |
|  | bta04931 | Insulin resistance | | 7 | 3.98E-02 |
|  | bta04724 | Glutamatergic synapse | | 7 | 4.29E-02 |
|  | bta04914 | Progesterone-mediated oocyte maturation | | 6 | 4.89E-02 |
| 35 | bta04115 | p53 signaling pathway | | 6 | 2.01E-03 |
|  | bta04917 | Prolactin signaling pathway | | 6 | 2.41E-03 |
|  | bta04066 | HIF-1 signaling pathway | | 6 | 7.36E-03 |
|  | bta04978 | Mineral absorption | | 4 | 1.81E-02 |
|  | bta05200 | Pathways in cancer | | 11 | 2.92E-02 |
|  | bta05168 | Herpes simplex infection | | 7 | 3.37E-02 |
|  | bta04713 | Circadian entrainment | | 5 | 3.47E-02 |
|  | bta04630 | Jak-STAT signaling pathway | | 6 | 4.29E-02 |
| 50 | bta00480 | Glutathione metabolism | | 4 | 3.89E-02 |
|  | bta04923 | Regulation of lipolysis in adipocytes | | 4 | 4.07E-02 |
| 140 | bta04010 | MAPK signaling pathway | | 8 | 4.96E-02 |

**Table S13.** Pathways analysis for the genes associated with FY of TD7, 35, 50, 140 and 280

| **DIM** | **accession number** | **KEGG pathway** | **No. Genes** | | **p-value** |
| --- | --- | --- | --- | --- | --- |
| 7 | bta04270 | Vascular smooth muscle contraction | | 10 | 2.60E-06 |
|  | bta04975 | Fat digestion and absorption | | 7 | 5.08E-06 |
|  | bta00592 | alpha-Linolenic acid metabolism | | 6 | 6.14E-06 |
|  | bta00591 | Linoleic acid metabolism | | 6 | 3.07E-05 |
|  | bta04972 | Pancreatic secretion | | 8 | 4.61E-05 |
|  | bta00590 | Arachidonic acid metabolism | | 7 | 7.86E-05 |
|  | bta00565 | Ether lipid metabolism | | 6 | 9.98E-05 |
|  | bta00564 | Glycerophospholipid metabolism | | 7 | 4.08E-04 |
|  | bta04713 | Circadian entrainment | | 6 | 2.84E-03 |
|  | bta04921 | Oxytocin signaling pathway | | 7 | 3.28E-03 |
|  | bta00350 | Tyrosine metabolism | | 4 | 7.41E-03 |
|  | bta00071 | Fatty acid degradation | | 4 | 7.94E-03 |
|  | bta04014 | Ras signaling pathway | | 8 | 9.13E-03 |
|  | bta04970 | Salivary secretion | | 5 | 9.57E-03 |
|  | bta00220 | Arginine biosynthesis | | 3 | 1.53E-02 |
|  | bta00830 | Retinol metabolism | | 4 | 1.86E-02 |
|  | bta04730 | Long-term depression | | 4 | 2.13E-02 |
|  | bta04022 | cGMP-PKG signaling pathway | | 6 | 2.17E-02 |
|  | bta04971 | Gastric acid secretion | | 4 | 3.56E-02 |
| 35 | bta05412 | Arrhythmogenic right ventricular cardiomyopathy (ARVC) | | 4 | 3.98E-02 |
| 50 | bta04540 | Gap junction | | 7 | 1.23E-03 |
|  | bta04966 | Collecting duct acid secretion | | 4 | 5.53E-03 |
|  | bta04721 | Synaptic vesicle cycle | | 5 | 1.02E-02 |
|  | bta04976 | Bile secretion | | 5 | 1.32E-02 |
|  | bta04145 | Phagosome | | 7 | 1.98E-02 |
|  | bta04923 | Regulation of lipolysis in adipocytes | | 4 | 3.79E-02 |
|  | bta04070 | Phosphatidylinositol signaling system | | 5 | 4.32E-02 |
| 140 | bta04024 | cAMP signaling pathway | | 9 | 4.33E-03 |
|  | bta04210 | Apoptosis | | 5 | 8.68E-03 |
|  | bta04972 | Pancreatic secretion | | 5 | 3.70E-02 |
| 280 | bta04060 | Cytokine-cytokine receptor interaction | | 7 | 4.00E-03 |
|  | bta04062 | Chemokine signaling pathway | | 6 | 9.27E-03 |

**Table S14.** Pathways analysis for the genes associated with MY of TD7, 35, 50, 140 and 280

| **DIM** | **accession number** | **KEGG pathway** | **No. Genes** | | **p-value** |
| --- | --- | --- | --- | --- | --- |
| 35 | bta04611 | Platelet activation | | 8 | 4.57E-03 |
|  | bta04974 | Protein digestion and absorption | | 6 | 1.17E-02 |
|  | bta04071 | Sphingolipid signaling pathway | | 6 | 4.58E-02 |
| 50 | bta04974 | Protein digestion and absorption | | 6 | 2.11E-03 |
| 140 | bta04740 | Olfactory transduction | | 25 | 1.87E-03 |

**Table S15.** Pathways analysis for the genes associated with PP of TD7, 35, 50, 140 and 280

| **DIM** | **accession number** | **KEGG pathway** | **No. Genes** | | **p-value** |
| --- | --- | --- | --- | --- | --- |
| 7 | bta04740 | Olfactory transduction | | 36 | 3.15E-05 |
|  | bta05218 | Melanoma | | 7 | 1.17E-03 |
|  | bta04024 | cAMP signaling pathway | | 10 | 5.44E-03 |
|  | bta04015 | Rap1 signaling pathway | | 10 | 8.91E-03 |
|  | bta04911 | Insulin secretion | | 6 | 1.14E-02 |
|  | bta04923 | Regulation of lipolysis in adipocytes | | 5 | 1.27E-02 |
|  | bta04914 | Progesterone-mediated oocyte maturation | | 6 | 1.51E-02 |
|  | bta04810 | Regulation of actin cytoskeleton | | 9 | 2.41E-02 |
|  | bta04550 | Signaling pathways regulating pluripotency of stem cells | | 7 | 2.75E-02 |
|  | bta05220 | Chronic myeloid leukemia | | 5 | 3.25E-02 |
|  | bta04725 | Cholinergic synapse | | 6 | 3.54E-02 |
| 35 | bta04978 | Mineral absorption | | 4 | 1.48E-02 |
|  | bta04114 | Oocyte meiosis | | 5 | 4.52E-02 |
| 50 | bta04721 | Synaptic vesicle cycle | | 5 | 1.53E-02 |
|  | bta03015 | mRNA surveillance pathway | | 5 | 4.65E-02 |
| 140 | bta05223 | Non-small cell lung cancer | | 6 | 6.53E-04 |
|  | bta04917 | Prolactin signaling pathway | | 6 | 2.30E-03 |
|  | bta00640 | Propanoate metabolism | | 4 | 4.07E-03 |
|  | bta05214 | Glioma | | 5 | 8.87E-03 |
|  | bta04650 | Natural killer cell mediated cytotoxicity | | 6 | 1.57E-02 |
|  | bta04260 | Cardiac muscle contraction | | 5 | 1.80E-02 |
|  | bta04012 | ErbB signaling pathway | | 5 | 2.28E-02 |
|  | bta04912 | GnRH signaling pathway | | 5 | 2.28E-02 |
|  | bta04068 | FoxO signaling pathway | | 6 | 2.51E-02 |
|  | bta04540 | Gap junction | | 5 | 2.55E-02 |
|  | bta05213 | Endometrial cancer | | 4 | 2.60E-02 |
|  | bta04910 | Insulin signaling pathway | | 6 | 2.81E-02 |
|  | bta04014 | Ras signaling pathway | | 8 | 2.82E-02 |
|  | bta05231 | Choline metabolism in cancer | | 5.00E+00 | 3.58E-02 |
|  | bta04730 | Long-term depression | | 4 | 3.78E-02 |
|  | bta04010 | MAPK signaling pathway | | 8 | 4.12E-02 |
|  | bta05412 | Arrhythmogenic right ventricular cardiomyopathy (ARVC) | | 4 | 4.82E-02 |
| 280 | bta04978 | Mineral absorption | | 5 | 3.13E-03 |
|  | bta04540 | Gap junction | | 6 | 7.79E-03 |
|  | bta01100 | Metabolic pathways | | 26 | 3.00E-02 |
|  | bta05134 | Legionellosis | | 4 | 4.45E-02 |
|  | bta00980 | Metabolism of xenobiotics by cytochrome P450 | | 4 | 4.85E-02 |

**Table S16.** Pathways analysis for the genes associated with PY of TD7, 35, 50, 140 and 280

| **DIM** | **accession number** | **KEGG pathway** | **No. Genes** | | **p-value** |
| --- | --- | --- | --- | --- | --- |
| 7 | bta04921 | Oxytocin signaling pathway | | 10 | 4.93E-03 |
|  | bta05410 | Hypertrophic cardiomyopathy (HCM) | | 7 | 8.58E-03 |
|  | bta05414 | Dilated cardiomyopathy | | 7 | 1.20E-02 |
|  | bta05412 | Arrhythmogenic right ventricular cardiomyopathy (ARVC) | | 6 | 1.45E-02 |
|  | bta04260 | Cardiac muscle contraction | | 6 | 3.24E-02 |
| 35 | bta04750 | Inflammatory mediator regulation of TRP channels | | 6 | 6.49E-03 |
|  | bta04071 | Sphingolipid signaling pathway | | 6 | 1.17E-02 |
|  | bta01100 | Metabolic pathways | | 23 | 1.86E-02 |
|  | bta04932 | Non-alcoholic fatty liver disease (NAFLD) | | 6 | 3.54E-02 |
|  | bta04744 | Phototransduction | | 3 | 3.74E-02 |
|  | bta04920 | Adipocytokine signaling pathway | | 4 | 4.69E-02 |
|  | bta00590 | Arachidonic acid metabolism | | 4 | 4.85E-02 |
| 50 | bta05168 | Herpes simplex infection | | 9 | 3.37E-03 |
|  | bta05162 | Measles | | 7 | 9.99E-03 |
|  | bta05412 | Arrhythmogenic right ventricular cardiomyopathy (ARVC) | | 5 | 1.02E-02 |
|  | bta05164 | Influenza A | | 7 | 2.58E-02 |
|  | bta04512 | ECM-receptor interaction | | 5 | 2.71E-02 |
| 140 | bta05134 | Legionellosis | | 4 | 8.95E-03 |
|  | bta04145 | Phagosome | | 5 | 3.09E-02 |
| 280 | bta05010 | Alzheimer's disease | | 6 | 8.56E-03 |
|  | bta04713 | Circadian entrainment | | 4 | 3.18E-02 |
|  | bta00230 | Purine metabolism | | 5 | 3.66E-02 |
|  | bta05030 | Cocaine addiction | | 3 | 4.77E-02 |
|  | bta04114 | Oocyte meiosis | | 4 | 4.77E-02 |

**Table S17.** Pathways analysis for the genes associated with SCS of TD7, 35, 50, 140 and 280

| **DIM** | **accession number** | **KEGG pathway** | **No. Genes** | | **p-value** |
| --- | --- | --- | --- | --- | --- |
| 7 | bta04270 | Vascular smooth muscle contraction | | 10 | 3.94E-03 |
|  | bta04921 | Oxytocin signaling pathway | | 11 | 4.69E-03 |
|  | bta04141 | Protein processing in endoplasmic reticulum | | 12 | 4.77E-03 |
|  | bta04720 | Long-term potentiation | | 6 | 2.74E-02 |
|  | bta05031 | Amphetamine addiction | | 6 | 3.07E-02 |
|  | bta04744 | Phototransduction | | 4 | 3.27E-02 |
| 35 | bta04071 | Sphingolipid signaling pathway | | 9 | 2.70E-03 |
|  | bta05169 | Epstein-Barr virus infection | | 8 | 9.24E-03 |
|  | bta04015 | Rap1 signaling pathway | | 11 | 1.05E-02 |
|  | bta04722 | Neurotrophin signaling pathway | | 8 | 1.25E-02 |
|  | bta04261 | Adrenergic signaling in cardiomyocytes | | 8 | 1.90E-02 |
|  | bta04725 | Cholinergic synapse | | 7 | 2.26E-02 |
|  | bta04914 | Progesterone-mediated oocyte maturation | | 6 | 3.12E-02 |
|  | bta04142 | Lysosome | | 7 | 4.03E-02 |
|  | bta04611 | Platelet activation | | 7 | 4.16E-02 |
|  | bta05210 | Colorectal cancer | | 5 | 4.26E-02 |
|  | bta04664 | Fc epsilon RI signaling pathway | | 5 | 4.47E-02 |
| 50 | bta04062 | Chemokine signaling pathway | | 12 | 4.42E-03 |
|  | bta05034 | Alcoholism | | 12 | 1.31E-02 |
|  | bta04740 | Olfactory transduction | | 37 | 1.53E-02 |
|  | bta04970 | Salivary secretion | | 6 | 4.67E-02 |
| 140 | bta04740 | Olfactory transduction | | 49 | 2.67E-09 |
| 280 | bta00350 | Tyrosine metabolism | | 4 | 4.79E-03 |
|  | bta00260 | Glycine, serine and threonine metabolism | | 4 | 5.14E-03 |
|  | bta00532 | Glycosaminoglycan biosynthesis - chondroitin sulfate / dermatan sulfate | | 3 | 1.25E-02 |
|  | bta00360 | Phenylalanine metabolism | | 3 | 1.38E-02 |
|  | bta00410 | beta-Alanine metabolism | | 3 | 3.43E-02 |
